# ^68^Ga-FAPI PET imaging monitors response to combined TGF-**β**R inhibition and immunotherapy in metastatic colorectal cancer

**DOI:** 10.1172/JCI170490

**Published:** 2024-01-04

**Authors:** Ke Li, Wei Liu, Hang Yu, Jiwei Chen, Wenxuan Tang, Jianpeng Wang, Ming Qi, Yuyun Sun, Xiaoping Xu, Ji Zhang, Xinxiang Li, Weijian Guo, Xiaoling Li, Shaoli Song, Shuang Tang

**Affiliations:** 1 Cancer Institute, Department of Nuclear Medicine, Fudan University Shanghai Cancer Center, Shanghai, China.; 2 Center for Biomedical Imaging, Fudan University, Shanghai, China.; 3 Shanghai Engineering Research Center of Molecular Imaging Probes, Shanghai, China.; 4 Department of Oncology and; 5 School of Clinical Medicine, Shanghai Medical College, Fudan University, Shanghai, China.; 6 Department of Critical Care Medicine, The First Affiliated Hospital of Harbin Medical University, Harbin, China.; 7 Department of Colorectal Surgery, Fudan University Shanghai Cancer Center, Shanghai, China.; 8 Department of Oncology, Shanghai Medical College, Fudan University, Shanghai, China.; 9 Department of Gastrointestinal Medical Oncology, Fudan University Shanghai Cancer Center, Shanghai, China.; 10 Signal Transduction Laboratory, National Institute of Environmental Health Sciences, Research Triangle Park, North Carolina, USA.

**Keywords:** Gastroenterology, Oncology, Cancer immunotherapy, Colorectal cancer, Diagnostic imaging

## Abstract

**BACKGROUND:**

Improving and predicting tumor response to immunotherapy remains challenging. Combination therapy with a transforming growth factor-β receptor (TGF-βR) inhibitor that targets cancer-associated fibroblasts (CAFs) is promising for the enhancement of efficacy of immunotherapies. However, the effect of this approach in clinical trials is limited, requiring in vivo methods to better assess tumor responses to combination therapy.

**METHODS:**

We measured CAFs in vivo using the ^68^Ga-labeled fibroblast activation protein inhibitor-04 (^68^Ga-FAPI-04) for PET/CT imaging to guide the combination of TGF-β inhibition and immunotherapy. One hundred thirty-one patients with metastatic colorectal cancer (CRC) underwent ^68^Ga-FAPI and ^18^F-fluorodeoxyglucose (^18^F-FDG) PET/CT imaging. The relationship between uptake of ^68^Ga-FAPI and tumor immunity was analyzed in patients. Mouse cohorts of metastatic CRC were treated with the TGF-βR inhibitor combined with KN046, which blocks programmed death ligand 1 (PD-L1) and CTLA-4, followed by ^68^Ga-FAPI and ^18^F-FDG micro-PET/CT imaging to assess tumor responses.

**RESULTS:**

Patients with metastatic CRC demonstrated high uptake rates of ^68^Ga-FAPI, along with suppressive tumor immunity and poor prognosis. The TGF-βR inhibitor enhanced tumor-infiltrating T cells and significantly sensitized metastatic CRC to KN046. ^68^Ga-FAPI PET/CT imaging accurately monitored the dynamic changes of CAFs and tumor response to combined the TGF-βR inhibitor with immunotherapy.

**CONCLUSION:**

^68^Ga-FAPI PET/CT imaging is powerful in assessing tumor immunity and the response to immunotherapy in metastatic CRC. This study supports future clinical application of ^68^Ga-FAPI PET/CT to guide precise TGF-β inhibition plus immunotherapy in CRC patients, recommending ^68^Ga-FAPI and ^18^F-FDG dual PET/CT for CRC management.

**TRIAL REGISTRATION:**

CFFSTS Trial, ChiCTR2100053984, Chinese Clinical Trial Registry.

**FUNDING:**

National Natural Science Foundation of China (82072695, 32270767, 82272035, 81972260).

## Abstract 2

68Ga-FAPI PET/CT imaging function as a non-invasive biomarker to assess tumor immunity and guide precise TGF-&beta; inhibition to sensitize metastatic colorectal cancer to immunotherapy.

## Graphical abstract

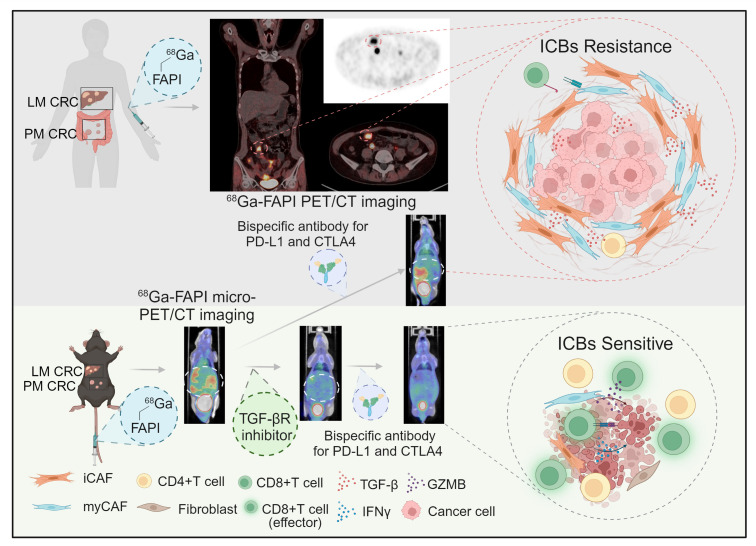

## Introduction

Colorectal cancer (CRC) is the third most common malignancy, and metastasis is the major cause of mortality (1). Liver or peritoneal metastasis is common among patients with CRC, leading to a poor prognosis and short overall survival (2, 3). Therefore, there is an urgent need to develop effective strategies to improve the prognosis of patients with metastatic CRC.

Immune checkpoint blockades (ICBs) bring hope for long-term survival of cancer patients. But only a few subgroups of patients benefit from ICB therapy. Currently, pembrolizumab is recommended as the first-line treatment for patients with metastatic CRC and microsatellite instability–high or deficient mismatch repair (MSI-H/dMMR) tumors (4). However, only 10%–15% of patients with CRC with MSI-H/dMMR show relatively high immunogenicity to benefit from ICBs (5, 6). Moreover, approximately 30%–40% of patients with CRC undergoing curative resection of the primary tumor develop metastases in subsequent years (7), and metastatic CRC responds even worse to ICBs. Thus, improving the therapeutic response and selecting patients who can benefit from immunotherapy are crucial to improve the overall survival of patients with metastatic CRC.

Cancer-associated fibroblasts (CAFs), one of the major components of the tumor microenvironment (TME), remodel the extracellular matrix to regulate various biological behaviors related to tumor immunity and metastasis (8, 9). Notably, transforming growth factor-β (TGF-β) released by cancer cells and CAFs is central to immune suppression within the TME, and contributes to tumor immune evasion and poor responses to cancer immunotherapy (10). TGF-β released in the TME acts as a chemoattractant factor for fibroblasts to induce the formation of CAFs (11). TGF-β–SMAD signaling can function with ERK/MAPK and AKT-mTOR signaling to promote the activation and myofibroblast differentiation of CAFs (10). Moreover, TGF-β drives immune evasion in genetically reconstituted colon cancer metastasis (12). Therefore, inhibition of TGF-β signaling has been evaluated in multiple clinical trials as a major avenue to enhance the efficacy of cancer immunotherapies. Several preclinical studies have explored the combination of TGF-β and PD-1/PD-L1 inhibition as cancer therapy with near-uniform positive results across a wide range of tumor types (12–19). However, progress has been difficult as this approach began to emerge in clinical trials, with most trials failing to recapitulate the success observed in animal models (20). This is likely due to adverse effects and drug toxicities that limited the effective dose and duration of combined TGF-β inhibition and ICBs (21–24). Caution may be required in advancing combination drugs targeting TGF-β and PD-1/PD-L1 without a guiding biomarker (20). Therefore, accurate predictive biomarkers to identify the patients most likely to derive clinical benefit from the combination therapy of TGF-β and ICBs are required. Current methods like molecular pathological staining can be used to detect fibroblast activation protein-α (FAP-α) in tumors collected by biopsy or surgery. But these invasive approaches are limited by the high heterogeneity and dynamic changes of CAFs in different metastatic tumor lesions during cancer progression. Therefore, a noninvasive and whole-body detection method to guide TGF-β inhibition is essential to improve clinical implications of the combined TGF-β and ICB strategy to treat cancer.

Positron emission tomography/computed tomography (PET/CT) is an FDA-approved full-body modality for molecular imaging (25). Using fluorodeoxyglucose (FDG) as the tracer to detect tumor glycolysis, ^18^F-FDG PET/CT imaging is routinely used in clinical practice to detect and diagnose tumors (26). Moreover, development of radioactive molecular probes may enable PET imaging to monitor cancer therapeutic responses to ICBs and to select potential beneficiaries for treatments (25). For instance, ^89^Zr-atezolizumab PET imaging for the detection of tumor PD-L1 expression is a noninvasive approach for assessing clinical responses of cancer to PD-L1 blockade (27). CD8^+^-targeted PET imaging of tumor-infiltrating T cells, such as ^89^Zr-Df-IAB22M2C and ^89^ZED88082A PET imaging, can monitor the complex dynamics of CD8^+^ T cells in the context of ICBs, and may predict early response to immunotherapy (28, 29). Granzyme B PET imaging to detect granzyme B secreted by effector CD8^+^ T cells during immune responses can serve as an in vivo biomarker of early response to immunotherapy (30, 31). These PET imaging probes show significant potential for monitoring tumor responses to ICB treatment alone. Furthermore, FAP-α, a membrane serine protease that is exclusively expressed in type A CAFs (32, 33), is overexpressed in 85%–90% of CRC cases but is undetectable in normal tissues (34, 35). Therefore, FAP-α is considered as a proper diagnostic target for detection of multiple solid tumors, especially for metastatic CRC (34, 35). Gallium-68–labeled fibroblast activation protein inhibitor-04 (^68^Ga-FAPI-04) for PET imaging has shown promising value in cancer detection. Particularly in advanced CRC, ^68^Ga-FAPI has high tumor uptake and can easily delineate tumor boundaries (36), thus serving as a powerful method for the detection of colorectal liver or peritoneal metastases in vivo (37–40). However, the potential of ^68^Ga-FAPI imaging for predicting or monitoring cancer therapeutic responses has not been evaluated. ^68^Ga-FAPI imaging detects CAFs that are regulated by TGF-β signaling and shape the immunosuppressive TME, suggesting that ^68^Ga-FAPI PET is uniquely useful in monitoring the metastatic CRC response to combination therapy of TGF-β inhibitor and ICBs.

In the present study, among patients with metastatic CRC in a clinical trial who underwent ^68^Ga-FAPI and ^18^F-FDG PET/CT imaging to detect tumor lesions (CFFSTS Trial, ChiCTR2100053984, Chinese Clinical Trial Registry), we determined that patients with colorectal peritoneal or liver metastasis displayed high tumor ^68^Ga-FAPI uptake, which was associated with significantly reduced tumor-infiltrated immune cells and a poor response to immunotherapy. Moreover, in preclinical mouse cohorts of peritoneal or liver metastatic CRC, ^68^Ga-FAPI micro-PET/CT imaging–guided precise usage of SB525334, a TGF-β receptor type 1 (TGF-βR) inhibitor, significantly improved tumor responses to KN046, a bispecific antibody that bifunctionally blocks CTLA-4 and PD-L1. Collectively, our results demonstrate the translational potential of ^68^Ga-FAPI PET/CT imaging in predicting or monitoring metastatic CRC response to immunotherapy and suggest that ^68^Ga-FAPI PET/CT may function as a noninvasive in vivo biomarker to guide precise TGF-β inhibition and improve clinical tumor response to immunotherapy. Therefore, this study supports future clinical trials that use ^68^Ga-FAPI PET/CT imaging as a noninvasive method to stratify and monitor patients with CRC for combined TGF-β signal inhibition and ICB therapy.

## Results

### Multi-tracer PET/CT imaging with ^68^Ga-FAPI and ^18^F-FDG identifies distinct heterogeneity of CAFs and glucose metabolism in patients with metastatic CRC.

**Figure 1:**
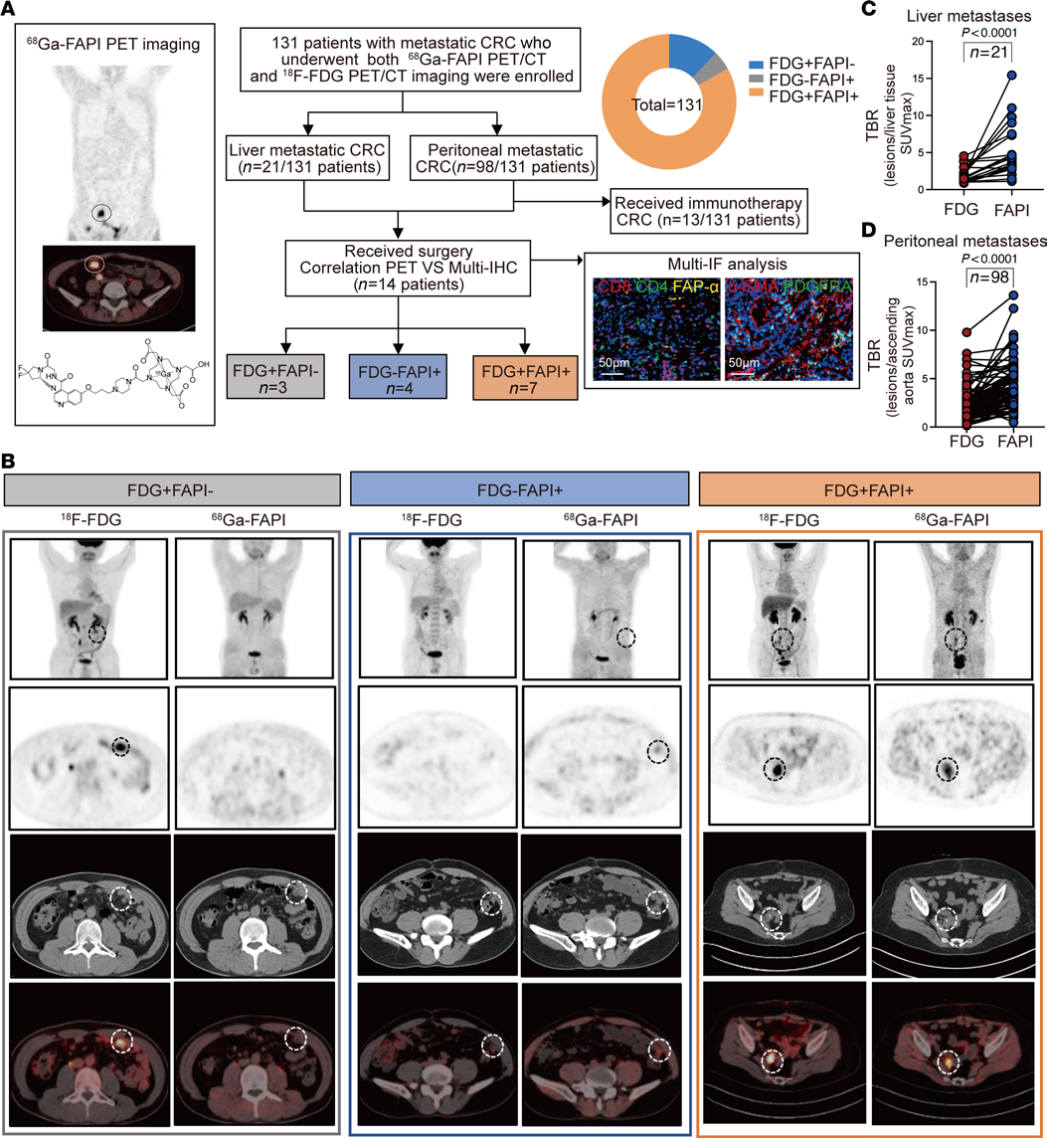
^68^Ga-FAPI PET/CT imaging adds value to ^18^F-FDG PET/CT imaging for detection of metastasis in patients with CRC. (**A**) Schematic flow of the patient selection process. In total, 131 patients with metastatic CRC who underwent both ^68^Ga-FAPI-04 and ^18^F-FDG PET/CT at the FUSCC were enrolled, including 21 patients with liver metastatic CRC, 98 with peritoneal metastatic CRC, and 12 with other metastases. Among them, 14 patients received surgery after imaging. The relationship between uptake of ^68^Ga-FAPI and tumor immunity was analyzed. Thirteen patients received immunotherapy after imaging. Patients who underwent ^68^Ga-FAPI PET/CT and ^18^F-FDG PET/CT were divided into 3 groups: FDG^+^FAPI^–^, FDG^–^FAPI^+^, and FDG^+^FAPI^+^. Proportions of each group are shown in the pie chart in the top right corner of the image. Scale bars: 50 μm. (**B**) Representative clinical ^68^Ga-FAPI PET/CT and ^18^F-FDG PET/CT images of patients with metastatic CRC. (**C**) Comparison of TBR SUVmax of ^68^Ga-FAPI and ^18^F-FDG in liver metastatic CRC tumors, *n* = 21. (**D**) Comparison of TBR SUVmax of ^68^Ga-FAPI and ^18^F-FDG in peritoneal metastatic CRC tumors, *n* = 98. All numerical data are presented as mean ± SEM. *P* < 0.0001 by Wilcoxon’s matched-pairs, signed-rank test (**C** and **D**).

High intestinal physiological uptake of ^18^F-FDG limits its diagnostic value in CRC management (41). Recently, the development of the PET tracer ^68^Ga-FAPI has shown promising results for the detection of primary and peritoneal metastatic CRC. To further evaluate the value of ^68^Ga-FAPI PET/CT imaging in the management of patients with metastatic CRC, we performed a clinical trial enrolling 131 patients with metastatic CRC to undergo both ^68^Ga-FAPI-04 and ^18^F-FDG PET/CT scans at the Fudan University Shanghai Cancer Center (FUSCC) (Figure 1A). Among the 131 patients with metastatic CRC, 109 patients (83.2%) showed high uptakes of both ^68^Ga-FAPI and ^18^F-FDG probes detected using PET/CT imaging, 16 patients (12.2%) showed high ^68^Ga-FAPI uptake but low ^18^F-FDG uptake, and 6 patients (4.6%) showed low uptake of ^68^Ga-FAPI but high uptake of ^18^F-FDG (the cutoff maximum standardized uptake value [SUVmax] was 2.0) (Figure 1, A and B). These results indicated high heterogeneity in both glucose metabolism and CAFs among patients with metastatic CRC.

Among the 131 patients with metastatic CRC who underwent ^68^Ga-FAPI and ^18^F-FDG PET/CT, there were 21 patients with liver metastases, 98 with peritoneal metastases, and 12 with other metastases. Despite the high heterogeneity of uptake values by ^68^Ga-FAPI and ^18^F-FDG PET/CT scans in all 131 patients, the ratio of the tumor to the background (TBR: SUVmax of lesion/SUVmax of background) of ^68^Ga-FAPI PET/CT in liver or peritoneal metastases was significantly higher than that of ^18^F-FDG PET/CT in both subgroups of patients with metastatic CRC (Figure 1, C and D). These results indicate the high sensitivity of ^68^Ga-FAPI PET/CT imaging for detecting the liver or peritoneal metastases of CRC. A summary of the clinical characteristics of the patients is presented in Supplemental Table 1 (supplemental material available online with this article; https://doi.org/10.1172/JCI170490DS1). Collectively, our observations indicated that ^68^Ga-FAPI PET/CT imaging added value to ^18^F-FDG PET/CT imaging in detecting metastases of patients with CRC and further suggested that CAFs are a potential target for metastatic CRC treatment.

### The SUVmax of ^68^Ga-FAPI PET/CT imaging negatively correlates with antitumor immunity in patients with metastatic CRC.

**Figure 2:**
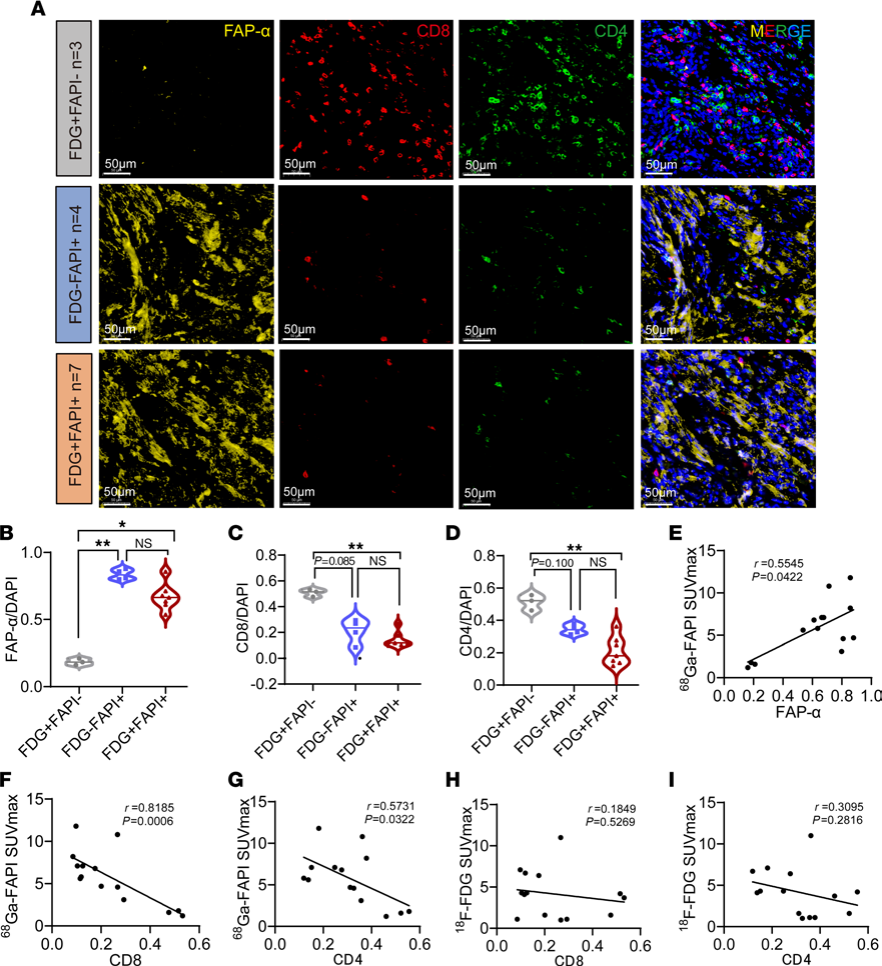
The SUVmax of ^68^Ga-FAPI PET/CT imaging negatively correlates with antitumor immunity in patients with metastatic CRC. (**A**) Representative multi-IHC images of the 3 groups in clinical metastatic CRC samples. FAP-α (yellow), CD8 (red), CD4 (green), and DAPI (blue) were used for staining of cell nuclei. Scale bars: 50 μm. (**B**–**D**) Quantification of staining for FAP-α, CD8, and CD4 for each group (FDG^+^FAPI^–^, *n* = 3; FDG^–^FAPI^+^, *n* = 4; FDG^+^FAPI^+^, *n* = 7). (**E**–**G**) Correlation between CD8, CD4, and FAP-α levels and SUVmax of ^68^Ga-FAPI in the 14 enrolled patients with metastatic CRC (by Pearson’s correlation analysis). (**H** and **I**) Correlation between CD8 levels, CD4 levels, and SUVmax of ^18^F-FDG in the 14 enrolled patients with metastatic CRC (by Pearson’s correlation analysis). All numerical data are presented as mean ± SEM. *P < 0.05, **P < 0.01 by 1-way ANOVA with Kruskal-Wallis test (**B**–**D**).

The important function of CAFs in tumor immune regulation led us to investigate the relationship between the tumor SUVmax of ^68^Ga-FAPI PET/CT imaging and antitumor immunity in patients with metastatic CRC (42). We used multi-immunofluorescence staining to analyze tumor-infiltrating immune cells in 14 patients with liver or peritoneal metastases who underwent surgery after both ^68^Ga-FAPI and ^18^F-FDG PET/CT scans at the FUSCC (Figure 1A). Notably, compared with tumors with high SUVmax by ^68^Ga-FAPI PET imaging, ^68^Ga-FAPI PET/CT–negative tumors showed low FAP-α protein expression and contained a significantly higher number of tumor-infiltrating CD8^+^ T cells and CD4^+^ T cells (Figure 2, A–D). However, no significant difference in tumor-infiltrating immune cells was observed in ^18^F-FDG PET/CT–negative tumors compared with ^18^F-FDG PET/CT–positive tumors (Figure 2, A–D). Moreover, the tumor SUVmax of ^68^Ga-FAPI exhibited a strong negative correlation with both tumoral infiltrated CD8^+^ and CD4^+^ immune cells using linear regression analysis and Pearson coefficients (Figure 2, E and F). Interestingly, Pearson coefficients showed that the degree of correlation between tumor SUVmax of ^68^Ga-FAPI and tumor-infiltrated CD8^+^ or CD4^+^ cells was comparable to the correlation between SUVmax of ^68^Ga-FAPI PET/CT and the expression of FAP-α (Figure 2G). In contrast, tumor uptake on ^18^F-FDG PET/CT exhibited no significant correlation with either tumor-infiltrated CD8^+^ or CD4^+^ immune cells (Figure 2, H and I). Collectively, the tumor SUVmax of ^68^Ga-FAPI PET/CT imaging was negatively associated with tumor infiltration of immune cells in patients with metastatic CRC. Therefore, ^68^Ga-FAPI PET/CT imaging may be helpful in screening potential beneficiaries of immunotherapy in patients with metastatic CRC.

### ^68^Ga-FAPI PET/CT as an imaging biomarker to assess therapeutic response to immunotherapy in patients with metastatic CRC.

**Figure 3:**
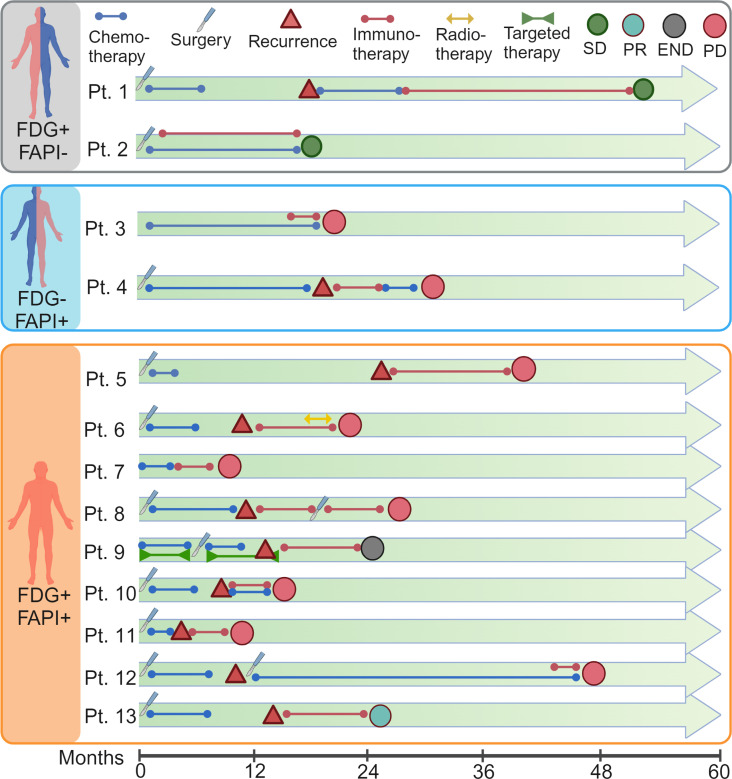
^68^Ga-FAPI PET/CT as an imaging biomarker to assess therapeutic response to immunotherapy in patients with metastatic CRC. Summary of clinical events and prognosis for the 13 patients with metastatic CRC who received immunotherapy after ^68^Ga-FAPI and ^18^F-FDG PET/CT. The 13 patients were divided into 3 groups: FDG^+^FAPI^–^, *n* = 2; FDG^–^FAPI^+^, *n* = 2; and FDG^+^FAPI^+^, *n* = 9. SD, stable disease; PR, partial response; PD, progressive disease; END, end of life. Created with BioRender (biorender.com).

To evaluate the potential of ^68^Ga-FAPI PET/CT imaging in predicting tumor responses to immunotherapy in patients with metastatic CRC, we enrolled and analyzed the prognosis of 13 patients with metastatic CRC who received immunotherapy (PD-1 or PD-L1 blockade) after ^68^Ga-FAPI PET/CT imaging in our clinical trial at the FUSCC (Figure 3). A summary of the clinical characteristics of the patients who received immunotherapy is presented in Table 1. Notably, 10 of 11 patients with a high SUVmax on ^68^Ga-FAPI PET/CT (patients 3–13 FDG^+/–^FAPI^+^) showed poor prognosis (progressive disease) after ICB therapy, and one died shortly thereafter. Interestingly, 2 patients with low SUVmax of ^68^Ga-FAPI PET/CT scan (patients 1 and 2 FDG^+^FAPI^–^) showed improved outcomes (stable disease) after immunotherapy. The χ^2^ test further showed that patients with metastatic CRC with a low SUVmax on ^68^Ga-FAPI PET/CT had significantly better clinical outcomes after immunotherapy (Table 2). However, the SUVmax of ^18^FDG-FAPI PET/CT was insignificant in predicting patient outcomes after immunotherapy (Table 3). These observations support our hypothesis that ^68^Ga-FAPI PET/CT imaging may help select potential patients with metastatic CRC for immunotherapy, indicating the necessity of a larger clinical trial using ^68^Ga-FAPI PET/CT imaging as a noninvasive in vivo method to select patients with metastatic CRC most likely to benefit from ICBs.

### ^68^Ga-FAPI PET/CT accurately monitors the dynamic changes of CAFs by TGF-β inhibition to assess tumor immunity and predict tumor response to ICBs in peritoneal metastatic CRC.

**Figure 4:**
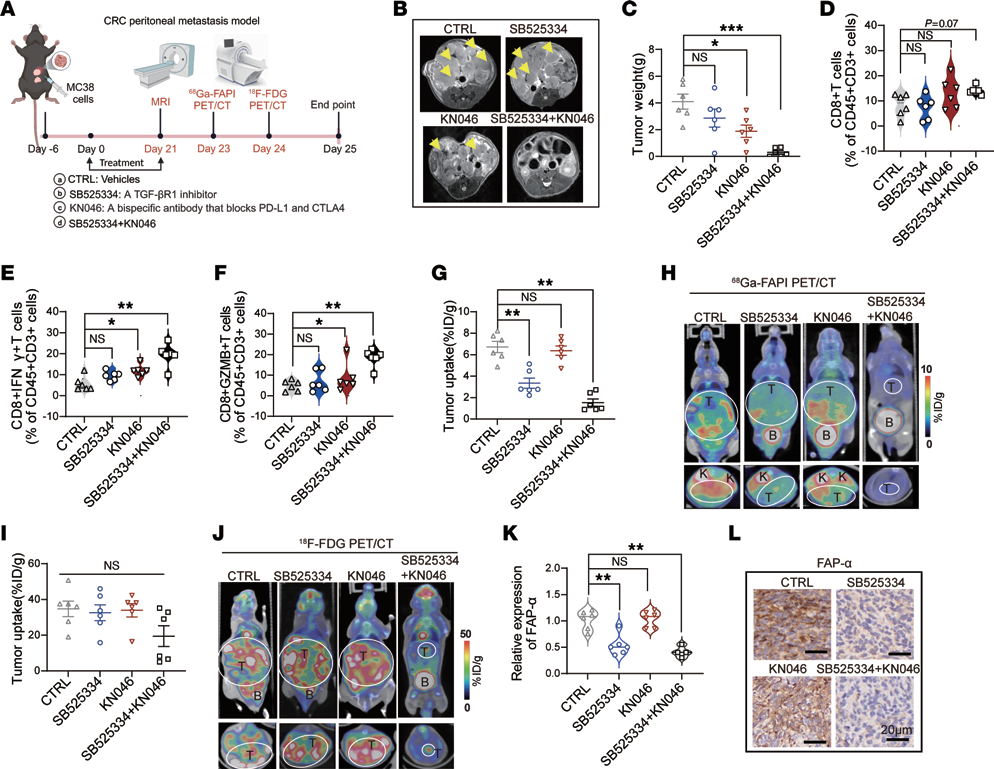
^68^Ga-FAPI micro-PET/CT and ^18^F-FDG micro-PET/CT scans to assess tumor response to combined therapy with TGF-βR inhibitor and ICB KN046 in mice with colorectal peritoneal metastasis. (**A**) Schematic of micro-MRI and PET imaging and treatment strategies in mice with MC38 peritoneal metastasis (4 groups, *n* = 6 per group). Created with BioRender. (**B**) Representative micro-MRI images of mice with peritoneal metastasis after the indicated treatments. Yellow arrows indicate tumor lesions. (**C**) Tumor weight of mice with MC38 peritoneal metastasis after the indicated treatments. (**D**–**F**) Proportion of CD8^+^ T cells, CD8^+^IFN-γ^+^ T cells, and CD8^+^GZMB^+^ T cells in CD45^+^CD3^+^ cells in peritoneal metastasis tumors harvested from mice in the 4 groups as determined using flow cytometry. (**G**) Quantified tumor uptake of ^68^Ga-FAPI in mice with peritoneal metastasis (*n* = 6 per group). (**H**) Representative ^68^Ga-FAPI micro-PET/CT images of mice with peritoneal metastasis after the indicated treatments. B, bladder; K, kidney; T, tumor. (**I**) Quantified tumor uptake of ^18^F-FDG in mice with peritoneal metastasis (*n* = 6 per group). (**J**) Representative ^18^F-FDG micro-PET/CT images of mice with peritoneal metastasis after the indicated treatments. (**K**) Quantified IHC staining of FAP-α in the tumors of mice with peritoneal metastasis after the indicated treatments. (**L**) Representative IHC staining of FAP-α in the tumors of mice with peritoneal metastasis after the indicated treatments. Scale bars: 20 μm. All numerical data are presented as mean ± SEM. *P < 0.05, **P < 0.01, *** *P* < 0.001, by 1-way ANOVA with Dunnett’s correct multiple-comparison test (**C**–**G**, **I**, and **K**).

Peritoneal metastasis is common in CRC patients, with poor prognosis and limited treatment options (43). The high uptakes of ^68^Ga-FAPI in PET/CT imaging in peritoneal metastasis of patients with CRC suggested that inhibition of CAFs by TGF-β signal inhibition may sensitize peritoneal metastatic CRC to ICBs. We assessed this hypothesis in 2 mouse models of MC38 or CT26 peritoneal metastatic CRC. Twenty-four mice with MC38 peritoneal metastatic CRC were randomly divided into 4 groups, then received vehicle control, SB525334 (a TGF-βR inhibitor) alone, KN046 (a bispecific antibody that blocks both PD-L1 and CTLA-4) alone, or combined SB525334 and KN046 treatment (Figure 4A). Magnetic resonance imaging (MRI) was performed 21 days after treatment to detect tumor. Notably, a reduction in tumor burden was observed after treatment with KN046 or KN046 combined with SB525334, whereas SB525334 alone was not effective in suppressing tumor growth compared with the control (Figure 4B). SB525334 in combination with KN046 showed the best efficacy in treating peritoneal metastatic CRC, achieving robust tumor remission in each mouse, as detected using micro-MRI (Figure 4B). Consistently, combined treatment with KN046 and SB525334 significantly decreased tumor weight, abdominal circumference, and the number of colorectal peritoneal metastases, together with significantly improved bloody ascites through peritoneal metastasis (Figure 4C and Supplemental Figure 1, A–C). The body weights of the MC38 tumor–bearing mice measured after the indicated treatments showed no significant changes among the 4 groups (Supplemental Figure 1D). These results indicate that the combined treatment with SB525334 and KN046 was effective in treating colorectal peritoneal metastasis in mice. In support of this, flow cytometric analyses and immunohistochemistry (IHC) revealed a significant increase in intratumoral CD8^+^ cytotoxic T cells (Supplemental Figure 1E for the flow cytometry gating strategy; Figure 4D; and Supplemental Figure 1F) and CD4^+^ T cells (Supplemental Figure 1, G and H) in tumors treated with combined SB525334 and KN046 therapy. Furthermore, intratumoral IFN-γ^+^CD8^+^ and granzyme B–positive CD8^+^ (GZMB^+^CD8^+^) T cells, as well as IFN-γ^+^CD4^+^ T cells, were also significantly increased by combined SB525334 with KN046 treatment (Figure 4, E and F, and Supplemental Figure 1I), suggesting that activated CD8^+^ and CD4^+^ T cells were increased in peritoneal metastasis by SB525334 combined with KN046. In addition, we obtained consistent results in a CT26 peritoneal metastatic CRC mouse model, in which combined treatment with SB525334 and KN046 significantly decreased peritoneal metastases and almost eliminated tumors in some mice (Supplemental Figure 2, A–E). This therapeutic effect was accompanied by a significant increase in intratumoral CD8^+^ and CD4^+^ T cells and a decrease in FAP-α in mice treated with the combination of SB525334 and KN046 (Supplemental Figure 2, F–I). Taken together, these results show that the TGF-βR inhibitor SB525334 effectively enhanced antitumor immunity and increased tumor response to KN046 in peritoneal metastatic CRC mice.

To evaluate whether ^68^Ga-FAPI PET/CT could accurately monitor the changes of CAFs by TGF-βR inhibitor, which enhanced antitumor immunity and sensitized peritoneal metastatic CRC to KN046 immune therapy, we performed ^68^Ga-FAPI micro-PET/CT after SB525334 and/or KN046 intervention to MC38 peritoneal metastatic CRC mice. Compared with the high SUVmax of ^68^Ga-FAPI PET in colorectal peritoneal metastases treated with PBS control, the tumor uptake of ^68^Ga-FAPI in mice treated with SB525334 was significantly decreased, especially in colorectal peritoneal metastases treated with SB525334 and KN046 (Figure 4, G and H). Consistent with this observation, SB525334 treatment significantly decreased FAP-α expression in the peritoneal metastasis models (Figure 4, G and H). These results suggest that ^68^Ga-FAPI PET/CT can accurately monitor the dynamic changes of CAFs by the TGF-βR inhibitor to assess tumor immunity and predict tumor response to ICBs in peritoneal metastatic CRC mice.

To further compare the accuracy of ^68^Ga-FAPI PET/CT imaging versus ^18^F-FDG PET/CT imaging (a widely used imaging modality for clinical cancer management) in monitoring the responses of colorectal peritoneal metastasis to immunotherapy, we also performed ^18^F-FDG micro-PET/CT imaging in the MC38 peritoneal metastasis mouse cohort 1 day after ^68^Ga-FAPI micro-PET/CT imaging and compared the results of these two PET probes (Figure 4A). Notably, ^18^F-FDG PET/CT imaging showed no significant differences in SUVmax among the 4 groups of tumors treated with SB525334 and/or KN046 compared with the PBS control (Figure 4, I and J). Combination therapy with SB525334 and KN046, which significantly decreased colorectal peritoneal metastasis in the mouse cohort (Figure 4, B and C), only slightly decreased the SUVmax of tumors on ^18^F-FDG PET/CT imaging (Figure 4, I and J). These results indicated that compared with ^18^F-FDG PET/CT, ^68^Ga-FAPI PET/CT detecting CAFs was more sensitive in monitoring tumor response to combined TGF-β inhibitor and ICBs in colorectal peritoneal metastasis. Interestingly, SB525334 alone significantly decreased CAFs but showed no significant impact on the growth of colorectal peritoneal metastasis (Figure 4, K and L). Notably, although ^68^Ga-FAPI PET/CT imaging accurately reflected the decrease of CAFs by TGF-βR inhibitor treatment and monitored the CAF inhibition–mediated synergistic effect on immunotherapy, ^18^F-FDG PET/CT was more sensitive in detecting the colorectal peritoneal metastasis upon TGF-βR inhibitor treatment (compare the imaging results in Figure 4I with those in Figure 4G).

Therefore, our results suggest that double-tracer PET/CT imaging integrating ^68^Ga-FAPI and ^18^F-FDG probes is necessary and feasible for the detection of tumor lesions and assessment of tumor response to immunotherapy in colorectal peritoneal metastasis.

### ^68^Ga-FAPI PET/CT guides scheduling of TGF-β inhibitor to optimize combination strategy with ICBs in peritoneal metastatic CRC.

**Figure 5:**
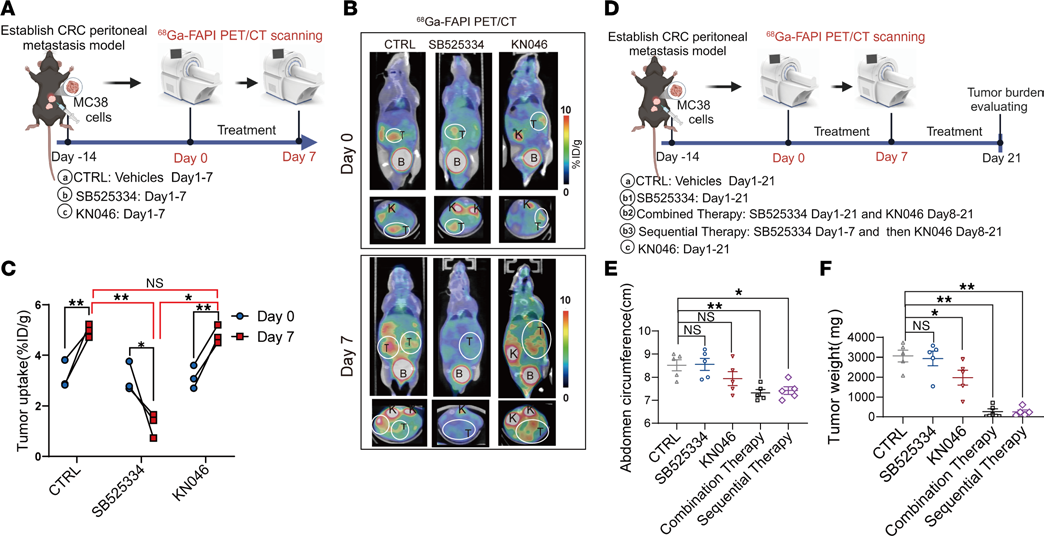
^68^Ga-FAPI micro-PET/CT and ^18^F-FDG micro-PET/CT imaging monitors responses to short-term TGF-β receptor inhibitor treatment in mice with colorectal peritoneal metastasis. (**A**) Schematic representation of micro-PET/CT imaging and treatment strategies in mice with MC38 peritoneal metastasis (3 groups: control group, *n* = 5; SB525334 group, *n* = 15; and KN046 group, *n* = 5). Created with BioRender. (**B**) Representative ^68^Ga-FAPI micro-PET/CT images of mice with peritoneal metastasis before (day 0) and after (day 7) the indicated treatments. B, bladder; K, kidney; T, tumor. (**C**) Quantified tumor uptake of ^68^Ga-FAPI in mice with peritoneal metastasis before (day 0, *n* = 3 per group) and after (day 7) the indicated treatments. (**D**) Schematic of micro-PET/CT imaging and treatment strategies in mice with MC38 peritoneal metastasis (5 groups, *n* = 5 per group). Created with BioRender. (**E**) Quantified abdomen circumference in tumor-bearing mice with peritoneal metastasis in the 5 groups. (**F**) Tumor weight of mice with MC38 peritoneal metastasis after the indicated treatments. All numerical data are presented as mean ± SEM. *P < 0.05, **P < 0.01, by 1-way ANOVA with Dunnett’s correct multiple-comparison test (**C**, **E**, and **F**).

Adverse effects and toxicities are the key factors limiting the clinical efficacy of combination therapy of TGF-β inhibitor and ICBs in clinical trials. To solve this clinical challenge, we investigated in a mouse cohort whether ^68^Ga-FAPI PET/CT imaging could help in deciding the schedule or combination strategy of TGF-β inhibitor and ICBs, in order to control drug side effects by reducing doses. First, we tested whether ^68^Ga-FAPI PET/CT imaging could detect the changes of CAFs upon short-term (7 days) TGF-β inhibitor treatment (Figure 5A). Mice with MC38 peritoneal metastatic CRC were randomly divided into 3 groups: vehicle control, SB525334 alone, and KN046 alone. ^68^Ga-FAPI micro-PET/CT was performed in mice with peritoneal metastatic CRC before treatment (day 0) and after 7-day treatment with TGF-βR inhibitor alone or KN046 alone (day 7) (Figure 5A). No significant differences in tumor ^68^Ga-FAPI uptake were observed among the 3 groups before TGF-βR inhibitor treatment (Figure 5B, top, and Figure 5C). Interestingly, ^68^Ga-FAPI PET/CT imaging showed notably decreased tumor uptake of ^68^Ga-FAPI upon short-term treatment with SB525334 alone in mice with MC38 peritoneal metastatic CRC, whereas short-term treatment with KN046 alone showed an insignificant effect (Figure 5B, bottom, and Figure 5C). These results indicated that ^68^Ga-FAPI PET/CT could detect the reduction of CAFs by short-term TGF-β inhibition in vivo.

Next, we compared therapeutic efficacy of different ^68^Ga-FAPI PET/CT imaging–guided combination strategies of TGF-βR inhibitor and KN046. Considering that ^68^Ga-FAPI PET/CT imaging detected significantly reduced ^68^Ga-FAPI tumor uptakes upon as short as 7-day TGF-βR inhibitor treatment in mice with peritoneal metastatic CRC, after that, we randomly divided those 7-day TGF-βR inhibitor–treated mice into 3 subgroups to receive different strategies of KN046 combination. From 8 days, mice of the combined therapy group received continuous TGF-βR inhibitor and started a combination of KN046 treatment to endpoint, mice of the sequential therapy group stopped TGF-βR inhibitor and switched to KN046 treatment alone to endpoint, and mice of the TGF-βR inhibitor–alone group received continuous TGF-βR inhibitor treatment without KN046 (Figure 5D). Interestingly, sequential therapy achieved a robust effect comparable to that of combined therapy for inhibiting the peritoneal tumor burden in mice. As compared with vehicle control, TGF-βR inhibitor–alone, or KN046-alone groups, mice of sequential therapy and combined therapy groups showed similarly and significantly decreased tumor weight of peritoneal metastases and abdomen circumference that reflects malignant ascites (Figure 5, E and F, and Supplemental Figure 1J). Additionally, none of the treatments had a significant effect on the body weight of the experimental mice (Supplemental Figure 1K). These findings suggested that short-term TGF-βR inhibitor treatment before immunotherapy is sufficient to improve metastatic CRC responses to ICBs, and ^68^Ga-FAPI PET/CT imaging can help optimize the sequential therapeutic strategy. Collectively, ^68^Ga-FAPI PET/CT imaging and changes of tumor ^68^Ga-FAPI PET signal help in deciding schedule of TGF-βR inhibitor in combination with ICBs.

### ^68^Ga-FAPI PET/CT imaging monitors the dynamic changes of CAFs by TGF-β inhibition to assess tumor response to ICBs in liver metastatic CRC.

Liver metastasis is the most common fatal disease of patients with CRC (44). Even though immunotherapy has proven successful in treating a subset of patients with CRC with MSI-H/dMMR, liver metastases diminish immunotherapy efficacy systemically in patients and preclinical models, as liver metastases result in an “immune desert” microenvironment either through macrophage-mediated T cell elimination (45) or through the “siphoning” of tumor antigen–specific CD8^+^ T cells into the liver (46). Our observations that the TGF-βR inhibitor SB525334 sensitized peritoneal metastatic CRC to the immune checkpoint inhibitor KN046 prompted us to further investigate whether TGF-βR inhibitor could also improve the response of colorectal liver metastasis to KN046.

**Figure 6:**
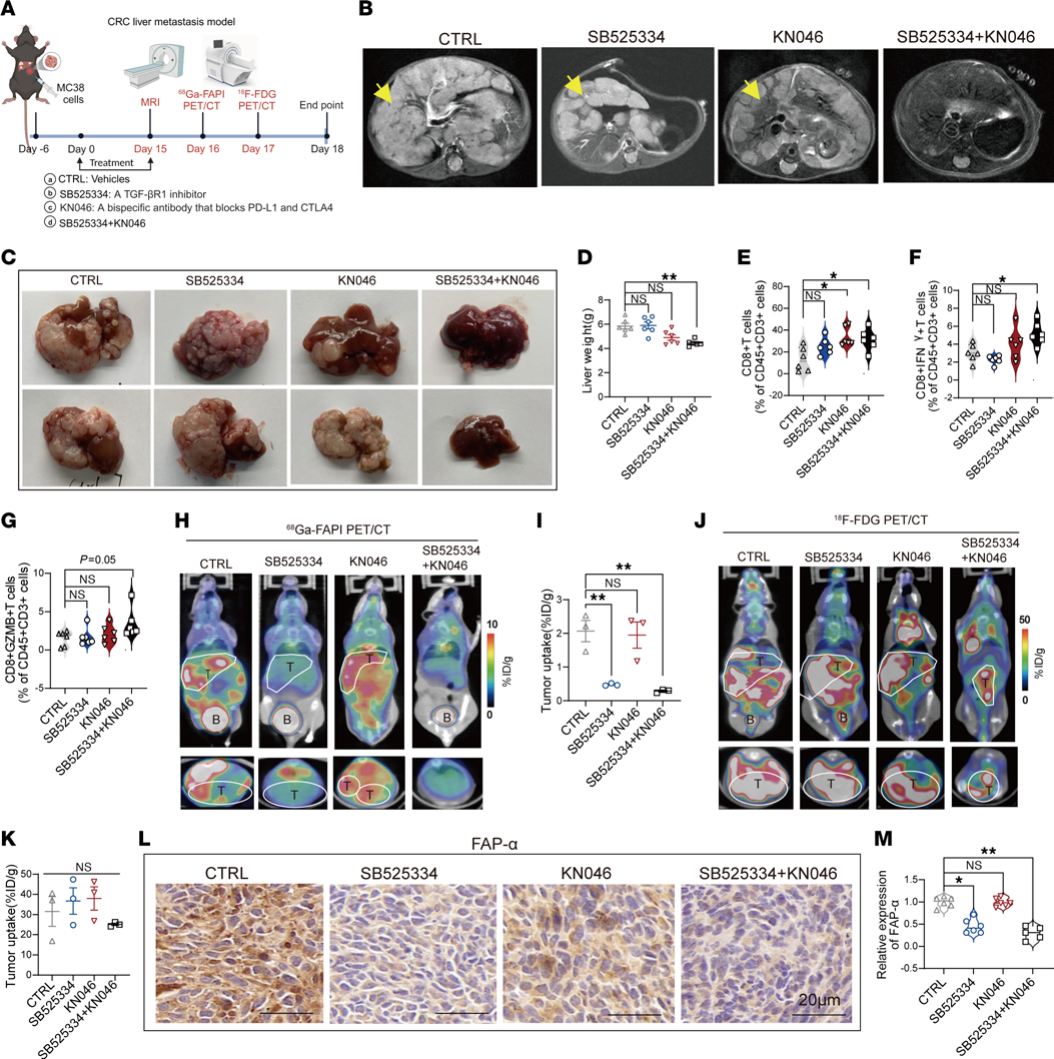
Using ^68^Ga-FAPI micro-PET/CT and ^18^F-FDG micro-PET/CT imaging to assess sensitization of colorectal liver metastases to ICB KN046 by TGF-β inhibition. (**A**) Schematic representation of MRI and PET imaging and treatment strategies in mice with MC38 liver metastasis (4 groups, *n* = 6 per group). Created with BioRender. (**B**) Representative micro-MRI images of mice with MC38 liver metastasis after the indicated treatments. Yellow arrows indicate tumor lesions. (**C**) Representative liver images of mice with liver metastasis after the indicated treatments. (**D**) Liver weights of mice with MC38 liver metastasis after the indicated treatments. (**E**–**G**) Proportion of CD8^+^ T cells, CD8^+^IFN-γ^+^ T cells, and CD8^+^GZMB^+^ T cells in CD45^+^ cells in liver metastasis harvested from mice of the 4 groups as measured using flow cytometry. (**H**) Representative ^68^Ga-FAPI micro-PET/CT images of mice with liver metastasis after the indicated treatments. B, bladder; K, kidney; T, tumor. (**I**) Quantified tumor uptake of ^68^Ga-FAPI in mice with liver metastasis (*n* = 3 per group). (**J**) Representative ^18^F-FDG micro-PET/CT images of mice with liver metastasis after the indicated treatments. (**K**) Quantified tumor uptake of ^18^F-FDG in mice with liver metastasis after the indicated treatments (*n* = 3 per group). (**L**) IHC staining of FAP-α in tumors of mice with liver metastasis after the indicated treatments. Scale bars: 20 μm. (**M**) Quantified IHC staining of FAP-α in tumors of mice with liver metastasis after the indicated treatments. All numerical data are presented as mean ± SEM. *P < 0.05, **P < 0.01 by 1-way ANOVA with Dunnett’s correct multiple-comparison test (**D**–**G**, **I**, **K**, and **M**).

Twenty-four mice with MC38 liver metastases were randomly divided into 4 groups and treated with KN046, SB525334, combined SB525334 and KN046, or control (Figure 6A). Micro-MRI was performed 15 days after treatment to detect the tumor burden of liver metastases. Compared with the PBS control, mice carrying MC38 liver metastasis partially responded to KN046 treatment alone, with a reduced tumor burden in some mice (Figure 6, B–D). SB525334 treatment alone was ineffective in treating mice with MC38 liver metastases (Figure 6, B–D). However, the combined treatment with SB525334 and KN046 significantly decreased the tumor number and burden of colorectal liver metastasis (Figure 6, B and C), along with notably decreased liver weight and abdominal circumference after 18 days (Figure 6D and Supplemental Figure 3A). None of the treatments had a significant effect on the body weight of the experimental mice (Supplemental Figure 3B). All these results demonstrated that the TGF-βR inhibitor SB525334 sensitized colorectal liver metastasis to KN046 in mice.

In line with the above observations, the combined SB525334 with KN046 therapy significantly increased intratumoral CD8^+^, IFN-γ^+^CD8^+^, and GZMB^+^CD8^+^ T cells and CD4^+^ and IFN-γ^+^CD4^+^ T cells in colorectal liver metastasis (Figure 6, E–G, and Supplemental Figure 3, C–F). These results suggest that the combined treatment with SB525334 and KN046 increased the activation of CD8^+^ and CD4^+^ T cells. Interestingly, KN046 alone also increased the intratumoral CD8^+^, CD4^+^, and IFN-γ^+^CD4^+^ T cells in colorectal liver metastasis, but it had no significant effect on IFN-γ^+^CD8^+^ and GZMB^+^CD8^+^ T cells (Figure 6, E–G, and Supplemental Figure 3, C–F), suggesting that although KN046 treatment increases the recruitment of CD4^+^ and CD8^+^ T cells into colorectal liver metastasis, it will not lead to activation of CD8^+^ T cells. In contrast, SB525334 alone did not increase intratumoral CD4^+^ and CD8^+^ T cells in liver metastases (Figure 6, E–G, and Supplemental Figure 3, C–F). Collectively, these results showed that the TGF-βR inhibitor SB525334 combined with KN046 induced more tumor-infiltrating activated T cells, which may contribute to the improved tumor response to KN046 in colorectal liver metastasis.

We next investigated whether ^68^Ga-FAPI PET/CT imaging could accurately reflect TGF-βR inhibitor–altered CAFs to assess SB525334-sensitized colorectal liver metastasis response to KN046. Consistent with the results in the colorectal peritoneal metastasis model, compared with the group treated with vehicle control or KN046 alone, the tumor uptake of ^68^Ga-FAPI in mice bearing liver metastasis was significantly lower in the group receiving SB525334 treatment, and the lowest tumor uptake was observed in the group treated with the combination of SB525334 and KN046 (Figure 6, H and I). In line with the results in the colorectal peritoneal metastasis model, ^18^F-FDG PET/CT imaging showed no significant differences in glucose uptake among the 4 groups (Figure 6, J and K), despite the accurate detection of tumor lesions in these mice (Figure 6, J and K). IHC staining of FAP-α demonstrated that the SB525334 treatment significantly decreased FAP-α expression in colorectal liver metastasis (Figure 6, L and M). Collectively, our observations indicated that for both peritoneal and liver metastasis of CRC, ^68^Ga-FAPI PET imaging is valuable in monitoring responses to therapy with combined TGF-β inhibitor and ICBs, and ^68^Ga-FAPI and ^18^F-FDG double-tracer PET/CT imaging is superior to single-probe PET imaging in immune-therapeutic management of colorectal liver metastasis.

### ^68^Ga-FAPI PET/CT imaging reflects abundance of both myofibroblastic CAFs and inflammatory CAFs in metastatic CRC.

Single-cell RNA sequencing (RNA-Seq) identified high heterogeneity among CAFs. To investigate whether ^68^Ga-FAPI PET/CT imaging can reflect tumor CAF subtypes in vivo, we stained metastatic CRC tumors that were detected by ^68^Ga-FAPI PET/CT or ^18^F-FDG PET/CT scan with α-smooth muscle actin (α-SMA), a marker for myofibroblastic CAFs (myCAFs) (47), and PDGFRA, a marker for the inflammatory subtype of CAFs (iCAFs) (48).

**Figure 7:**
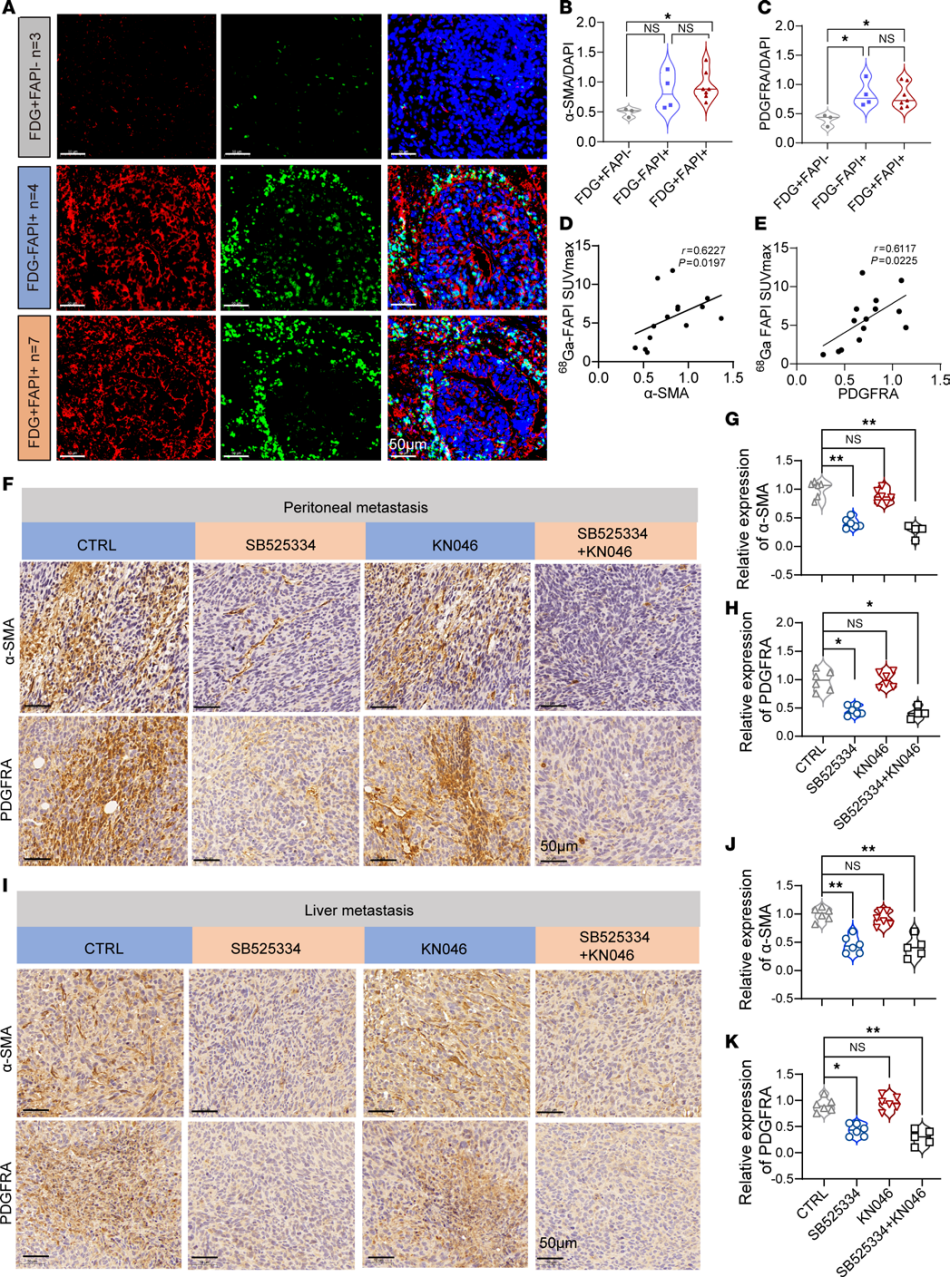
^68^Ga-FAPI PET/CT imaging reflects abundance of both myCAFs and iCAFs in metastatic CRC. (**A**) Multicolor immunofluorescence staining of α-SMA^+^ myCAFs (red) and PDGFRA^+^ iCAFs (green) in tumor tissues from the 14 patients with CRC who received surgery after ^68^Ga-FAPI and ^18^F-FDG PET/CT imaging at the FUSCC. Scale bars: 50 μm. (**B** and **C**) Quantified multi-immunofluorescence staining of α-SMA and PDGFRA in the tumors of 14 patients with CRC divided into 3 groups. (**D**) Positive correlation between α-SMA and ^68^Ga-FAPI SUVmax in the 14 clinical CRC samples (by Pearson’s correlation analysis). (**E**) Positive correlation between PDGFRA and ^68^Ga-FAPI SUVmax in the 14 clinical CRC samples (by Pearson’s correlation analysis). (**F**–**K**) Representative IHC staining and quantitative analyses of α-SMA and PDGFRA expression in both peritoneal metastasis and liver metastasis of mice with CRC treated with the indicated therapies. Scale bars: 50 μm. All numerical data are presented as mean ± SEM. *P < 0.05, **P < 0.01 by 1-way ANOVA with Kruskal-Wallis H test (**B** and **C**) and 1-way ANOVA with Dunnett’s correct multiple comparison test (**G**, **H**, **J**, and **K**).

**Figure 8:**
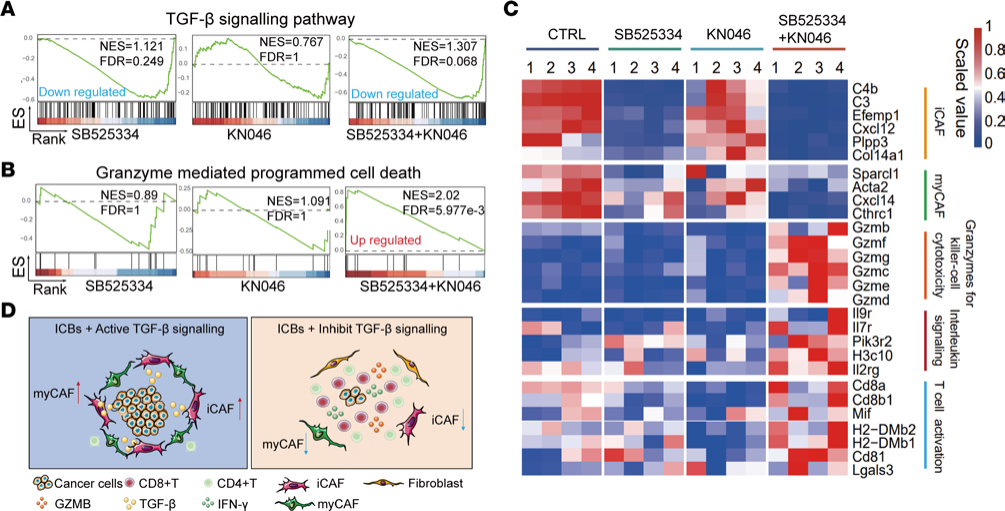
TGF-β inhibition suppresses CAFs and increases antitumor immunity in metastatic CRC tumors. (**A**) Gene set enrichment analysis (GSEA) of the TGF-β signaling pathway in liver metastasis treated with the indicated therapies compared with control (*n* = 4 per group). (**B**) GSEA of the granzyme-mediated programmed cell death pathway in liver metastasis treated with the indicated therapies compared with control (*n* = 4 per group). (**C**) Heatmap showing scaled normalized expression of marker genes in iCAFs, myCAFs, and granzymes for killer-cell cytotoxicity, interleukin signaling, and T cell activation pathways. (**D**) A working model showing that TGF-β inhibition reduces CAFs to improve antitumor immunity and increase efficacy of ICBs for cancer treatment.

We stained samples from patients with CRC who underwent ^68^Ga-FAPI PET/CT and ^18^F-FDG PET/CT with α-SMA for myCAFs and PDGFRA for iCAFs. Notably, ^68^Ga-FAPI PET/CT–positive tumors showed significantly higher expression of both α-SMA and PDGFRA than ^68^Ga-FAPI PET/CT–negative tumors (Figure 7, A–C), suggesting high abundance of both myCAFs and iCAFs in metastatic CRC tumors that uptake high ^68^Ga-FAPI. In contrast, PDGFRA and α-SMA expression did not differ between ^18^F-FDG PET/CT–positive and ^18^F-FDG PET/CT–negative patients (Figure 7, A–C). Moreover, SUVmax of ^68^Ga-FAPI PET/CT exhibited strong positive correlations with both α-SMA expression and PDGFRA expression in patients with colorectal peritoneal and liver metastases (Figure 7, D and E). These results suggest that ^68^Ga-FAPI PET/CT reflects the abundance of both myCAFs and iCAFs, but is unable to distinguish between CAF subtypes, in patients with metastatic CRC. Furthermore, the TGF-βR inhibitor SB525334 alone or in combination with KN046, which decreased TGF-β signaling as measured by phosphorylated SMAD2/3 (p-SMAD2/3) levels, effectively reduced expression of multiple markers for myCAFs (α-SMA, periostin, transgelin) and iCAFs (PDGFRA, CXCL12, IL-6) in both peritoneal and liver metastasis of CRC mice (Figure 7, F–K, and Supplemental Figure 4, A–E) (49). Finally, RNA-Seq on tumor tissues isolated from mice with MC38 liver metastasis revealed significantly downregulated TGF-β signaling in the SB525334 or the combined SB525334 and KN046 group compared with the control group (Figure 8A and Supplemental Figure 4F). Impressively, the combined SB525334 and KN046 group presented significantly upregulated granzymes, which are central factors in antitumor immunity (Figure 8B). A heatmap of significantly altered genes further showed that combined TGF-βR inhibitor and KN046 treatment decreased multiple gene markers for both iCAFs and myCAFs, accompanied by increased T cell activation, interleukin signaling, and granzymes for killer-cell cytotoxicity (Figure 8C). These results suggested that TGF-βR inhibitor suppressed both myCAFs and iCAFs in metastatic CRC tumors to increase antitumor immunity and tumor responses to KN046 (Figure 8D), which can be accurately detected by ^68^Ga-FAPI micro-PET/CT. Collectively, ^68^Ga-FAPI PET/CT is superior to ^18^F-FDG PET/CT in reflecting tumor CAF abundance, which correlates with the tumor response to immunotherapy in metastatic CRC.

## Discussion

Improving the efficacy of immunotherapy and noninvasively selecting patients who are most likely to respond to ICBs remain major clinical challenges in cancer treatment. Recent studies have highlighted the major role of CAFs in cancer immunotherapy resistance (13, 50, 51). Combined TGF-β inhibition and ICBs showed promising effects for cancer therapy in several preclinical models (12–19). However, this combination approach had unsatisfactory efficacy in most clinical trials (52), requiring biomarkers to guide precise inhibition of TGF-β to improve efficacy of immunotherapy. ^68^Ga-FAPI PET/CT targeting FAP-α has been used for the visualization of CAFs to detect tumor lesions in clinical settings (53); however, its value in assessing cancer response to immunotherapy is unknown. We determined that CRC patients with a high SUVmax of ^68^Ga-FAPI recruited notably fewer T cells into their tumor beds, which was associated with poor responses to immunotherapy. Moreover, in metastatic CRC mouse models, we detected a decrease of CAFs through the TGF-βR inhibitor SB525334, which significantly sensitized metastatic CRC to immunotherapy by improving the tumor immune microenvironment, and led to significantly reduced tumor uptakes of ^68^Ga-FAPI by PET/CT. Therefore, ^68^Ga-FAPI PET/CT imaging is a powerful noninvasive tool for assessing the CRC response to immunotherapy in metastatic CRC by detecting CAFs in vivo.

Patients with metastatic CRC with liver or peritoneal metastases respond poorly to ICB therapy. The combination of targeted therapies is considered the most promising strategy to improve the efficacy of cancer immunotherapy (54). Interestingly, although inhibition of TGF-β signaling that targets CAFs is a promising strategy to enhance efficacy of cancer immunotherapies, systemic adverse effects and the therapeutic index of TGF-β inhibitor need careful consideration (55). In clinical trials among patients with CRC, combined inhibition of TGF-β and PD-1/PD-L1 signaling caused multiple adverse effects, including pneumonitis, nausea, pruritus, rash, adrenal insufficiency, and hepatic impair (56–58). Moreover, TGF-β inhibitors can have both pro-tumorigenic and anti-tumorigenic effects (55, 59), as TGF-β also functions as a potent tumor suppressor by inducing growth inhibition and apoptosis in premalignant cells (55). Therefore, the precise guidance of the use of TGF-β inhibitor has become a crucial challenge for its clinical implications to improve immunotherapy efficacy. Using mouse cohorts, we showed that ^68^Ga-FAPI micro-PET/CT accurately detected the reduction of CAF abundances by TGF-βR inhibitor, and combined TGF-βR inhibitor and KN046 achieved significant tumor inhibition in colorectal liver and peritoneum metastasis. Decreased tumor FAPI signal detected by ^68^Ga-FAPI PET/CT after as few as 7 days of TGF-βR inhibitor SB525334 treatment optimized the combination of TGF-βR inhibitor and ICBs to a sequential strategy that involved stopping the TGF-βR inhibitor and switching to KN046, which can shorten TGF-βR inhibitor treatment to reduce adverse effects and achieve similarly notable therapeutic effects with the combination strategy. Therefore, ^68^Ga-FAPI PET/CT can function as a noninvasive biomarker to guide the delivery and schedule of TGF-β inhibitors to sensitize metastatic CRC to immunotherapy.

Recent studies have compared the diagnostic sensitivity of ^68^Ga-FAPI PET/CT and ^18^F-FDG PET/CT in primary and recurrent CRC (60), and reported that ^68^Ga-FAPI PET/CT achieved notably higher sensitivity and specificity in the detection of primary lesions and malignancies that metastasize to the peritoneum or liver (39). However, the value of ^68^Ga-FAPI and ^18^F-FDG PET/CT in predicting or monitoring cancer responses to immunotherapy remains largely unknown. Our study revealed that ^68^Ga-FAPI PET/CT imaging is superior to ^18^F-FDG PET/CT in assessing the response of metastatic CRC to ICBs. Specifically, ^68^Ga-FAPI PET/CT imaging accurately detected the decrease of CAFs by TGF-βR inhibitor in colorectal liver and peritoneal metastasis. High tumor uptake of ^68^Ga-FAPI is strongly associated with reduced tumor-infiltrating immune cells and function, leading to poor prognosis in patients with metastatic CRC. Notably, although ^18^F-FDG PET/CT showed limited value in reflecting changes in CAFs, and it was associated with improved tumor immunity. However, ^18^F-FDG PET/CT detected tumors with low CAFs, which may be missed by ^68^Ga-FAPI PET/CT. Therefore, dual probes targeting both ^68^Ga-FAPI and ^18^F-FDG in PET/CT are recommended for CRC management.

In conclusion, ^68^Ga-FAPI PET/CT imaging as a noninvasive tool for detecting CAFs can accurately reflect tumor immunity and monitor the metastatic CRC response to immunotherapy in vivo. We also provided preclinical evidence that TGF-β receptor inhibitor suppresses CAFs effectively sensitized colorectal liver and peritoneum metastasis to KN046 that blocking both PD-L1 and CTLA-4, and the tumor responses can be accurately measured in vivo by ^68^Ga-FAPI PET/CT imaging. ^68^Ga-FAPI PET/CT imaging assists in selecting patients with metastatic CRC who can benefit from immunotherapy, guiding precise scheduling of TGF-β inhibition to optimize the combination strategy with immunotherapy. Our study suggests a strategy of using ^68^Ga-FAPI PET/CT imaging–guided precise TGF-β inhibition to sensitize metastatic CRC to immunotherapy, and highlights the necessity of using double-tracer PET/CT imaging with ^68^Ga-FAPI and ^18^F-FDG for the management of patients with CRC.

## Methods

### Patient inclusion in the clinical trial.

This study retrospectively included 131 patients with metastatic CRC who underwent ^68^Ga-FAPI PET/CT and ^18^F-FDG PET/CT imaging between July 2020 and October 2023 at the FUSCC. The inclusion criteria were as follows: (a) liver or peritoneal metastatic CRC diagnosis based on the Chinese Society of Clinical Oncology guidelines; (b) underwent ^68^Ga-FAPI PET/CT and ^18^F-FDG PET/CT with an interval of less than 5 days; and (c) received standard treatment including surgery, chemotherapy, and/or immunotherapy. The exclusion criteria were as follows: (a) newly diagnosed patients with CRC with no metastasis and (b) multiple metastases other than the liver or peritoneum. Detailed patient information is presented in Supplemental Table 1.

### Animal models.

Six-week-old male wild-type C57BL/6 mice were housed in a pathogen-free facility. For peritoneal metastasis of CRC models, MC38 or CT26 CRC cells (2 × 10^5^ cells) were intraperitoneally injected into the abdominal cavity of 6-week-old male C57BL/6 or BALB/c mice. For liver metastasis of CRC models, MC38 cells (2 × 10^5^ cells) suspended in 40 μL PBS were injected into the inferior hemispleen of each 6-week-old C57BL/6 mouse. KN046 (Alphamab Oncology) was injected intraperitoneally at a dose of 10 mg/kg twice a week. The TGF-β receptor I (ALK5) inhibitor was SB525334 (Selleckchem) dissolvaed in CMC-Na and was given at 20 mg/kg dose by oral gavage daily. Our study exclusively examined male mice. It is unknown whether the findings are relevant to female mice.

### Synthesis of ^68^Ga-FAPI and ^18^F-FDG.

At our center, we use the Explora FDG4 module with a cyclotron (CTI RDS Eclipse ST, Siemens, Knoxville, Tennessee, USA) to automatically produce ^18^F-FDG (61). DOTA-FAPI-04 was obtained commercially (Jiangsu Huayi Technology Co. Ltd.) and radiolabeled with ^68^Ga according to the protocol published by Lindner et al. (62). DOTA-FAPI-04 and ^68^Ga solution eluted from ^68^Ge/^68^Ga generator (IGG100, Eckert & Ziegler) were mixed with NaAc (0.5 mL). The pH was maintained at approximately 4.5, and the mixture was heated at 100°C for 10 minutes (56). The radiochemical purities of FDG and FAPI were greater than 95%.

### Small-animal PET/CT scanning and MRI procedure.

Mice were fasted for 6 hours before ^18^F-FDG was administered intravenously via the tail vein, but not before ^68^Ga-FAPI tracer injection. Mice bearing xenografted MC38 tumors were placed on a heating pad (25°C) and were anesthetized using O_2_/isoflurane mixture (1%–2.5% isoflurane, 0.6–1 L/min O_2_). The FDG or FAPI micro-PET/CT scan was initiated 60 minutes and 30 minutes, respectively, after administration of the tracer (0.74–1.85 MBq). Immediately after CT scanning, 10-minute PET acquisition was performed using a Siemens Inveon PET/CT. Inveon Research Workplace 4.2 was used to analyze images, and regions of interest were applied to estimate the tumor uptake.

MRI was performed on a Bruker Biospec 70/20 USR scanner (Germany). Structural T1-weighted, T2-weighted, and fluid-attenuated inversion recovery (FLAIR) sequences were used for the detection of peritoneal and liver metastases. Rapid acquisition with relaxation enhancement (RARE) with a fat saturation sequence was used to acquire T2-weighted images with the following parameters: repetition time (TR) = 2,500 milliseconds (ms), echo time (TE) = 30 ms, RARE factor = 8, field of view (FOV) = 35 × 35 mm, matrix = 256 × 256, slice thickness = 1 mm, and scanning time = 5 minutes 20 seconds. A fast low-angle shot (FLASH) with a fat saturation sequence was used to acquire T1-weighted images with the following parameters: TR = 280 ms, TE = 2.9 ms, flip angle = 50°, FOV = 35 × 35 mm, matrix = 192 × 192, slice thickness = 1 mm, and scanning time = 3 minutes 35 seconds. The T2-FLAIR scan parameters were as follows: FOV = 35 × 35 mm, matrix = 256 × 256, slice thickness = 1 mm, TR = 10,000 ms, TE = 36 ms, inversion time = 2,000 ms, and scanning time = 4 minutes.

### Cell cultures.

The mouse CRC cell line MC38 was provided by Yanlei Ma at the FUSCC. CT26 cells were cultured in RPMI 1640 medium, and MC38 cells were cultured in Dulbecco’s modified Eagle medium (DMEM) with 10% FBS at 37°C in a humidified 5% CO_2_ atmosphere. All cells were authenticated and tested for mycoplasma.

### Immune cell isolation from tumors.

Tumors were collected and mechanically minced and incubated in digested buffer (DNase I [50 μg/mL; MilliporeSigma], collagenase [2 mg/mL; MilliporeSigma], DMEM, FBS, and penicillin/streptomycin) for 30 minutes at 37°C. The digested cells were mashed through 70 μm filters (BD Falcon) and then washed in FACS buffer (PBS with 0.5% endotoxin-free FBS, 2 mM EDTA, and 25 mM HEPES). The cells were collected and analyzed using flow cytometry.

### Flow cytometry.

The filtered tumor tissue cells were blocked with an anti-CD16/32 antibody (catalog 101319; 1 μg per 106 cells in 100 μL dilution buffer; BioLegend) and stained with indicated surface antibodies. Dead cells were marked using a Live/Dead Fixable Aqua dye (catalog L34965, Thermo Fisher Scientific). Fluorochrome-conjugated or biotinylated antibodies and their source, dilution information, and manufacturer are as follows: PerCP-Cy5.5–anti–mouse CD45 (1:200; clone 30-F11, catalog 103131, BioLegend), PE–Dazzle anti–mouse CD3ε (1:200; clone 145-2C11, catalog 100347, BioLegend), APC-Cy7–anti–mouse CD4 (1:200; clone RM4-5, catalog 100526, BioLegend), Alexa Fluor 700–anti–mouse CD8a (1:200; clone 53-6.7, catalog 100730, BioLegend), PE–anti–mouse IFN-γ (1:200; clone XMG1.2, catalog 505808, BioLegend), PE-Cyanine7–anti–mouse granzyme B (1:200; clone NGZB, catalog 25-8898-82, Invitrogen). Intracellular antibodies were added after fixation (catalog 420801, BioLegend) and permeabilization (catalog 421002, BioLegend), according to the manufacturer’s instructions. A Beckman Coulter CytoFLEX was used for our analysis, and FlowJo (version 10.8.1, Tree Star) was used for data analysis. Supplemental Figure 1E details the flow cytometry gating strategy.

### Histology and IHC and histopathological quantifications.

Tissues were harvested and fixed in 4% paraformaldehyde. Antibodies against CD4 (rabbit, reactive with human; 1:300; ab133616, Abcam), CD4 (rabbit, reactive with mouse; 1:400; ab183685, Abcam), CD8a (rabbit, reactive with human; 1:300; ab237709, Abcam), CD8a (rabbit, reactive with mouse; 1:500; ab217344, Abcam), FAP-α (rabbit; 1:300; ab218164, Abcam), α-SMA (rabbit; 1:250; 19245S, Cell Signaling Technology), PDGFRA (rabbit; 1:300; ab203491, Abcam), periostin (rabbit; 1:50; 19899-1-AP, Proteintech), transgelin (rabbit; 1:100; 10493-1-AP, Proteintech), CXCL12 (rabbit; 1:100; 17402-1-AP, Proteintech), and IL-6 (mouse; 1:200; ab9324, Abcam) were used for staining overnight at 4°C. Histological and IHC images were obtained using the Dako Autostainer Link 48 system (Agilent). Three fields on each slide were randomly selected for quantitative analysis. An IHC score (range 0–8) was assigned as follows: The staining intensity was scored on a scale of 0–3: 0, negative; 1, weak; 2, moderate; and 3, strong. The percentage of positive cells in the tissue was scored on a scale of 0–5: 0, no staining; 1, 1%–10% positive; 2, 11%–25% positive; 3, 26%–50% positive; 4, 51%–75% positive; 5, 76%–100% positive. The IHC score was the staining intensity score plus the percentage of positive cells score.

Multicolor immunofluorescence was performed using the Opal 4-Color Manual IHC Kit (abs50012, Absin) according to the manufacturer’s protocol. Briefly, sections were subjected to microwave-induced antigen retrieval in EDTA buffer (pH 8.0), and endogenous peroxidase was blocked in 0.3% hydrogen peroxide in methanol. Sections were then washed in PBST, blocked with 5% goat serum in PBS for 10 minutes, and incubated with the primary antibody for 1 hour. A horseradish peroxidase–labeled goat anti-rabbit/mouse secondary antibody was used and developed with a fluorescent dye. For multiple fluorescent staining, sections were processed starting from the antigen retrieval step to remove binding antibodies and then incubated with another primary antibody. This was repeated until all the antigens were stained. The following antibody sequences were used: (a) CD4 (rabbit; 25229S, Cell Signaling Technology)–TSA 520, CD8 (rabbit; ab237709, Abcam)–TSA 570, and FAP-α (rabbit; ab218164, Abcam)–TSA 620; and (b) α-SMA (rabbit; 19245S, Cell Signaling Technology)–TSA 520 and PDGFRA (rabbit; 3174S, Cell Signaling Technology)–TSA 570. Finally, the sections were counterstained with DAPI and mounted in a glycerol and gelatin mounting medium. Tissue sections were imaged using an A1 scanning confocal microscope (Nikon). Confocal images were captured with a ×20 or ×10 objective, and image data were collected using NIS Elements (v4.50.00, Nikon).

### Western blotting.

Western blotting assays for tumor tissue proteins were performed according to the protocols provided by Abcam. Primary antibodies against p-SMAD2/3 (rabbit; 8828S, Cell Signaling Technology), SMAD2/3 (rabbit; 8685S, Cell Signaling Technology), and GAPDH (rabbit; 2118S, Cell Signaling Technology) were used for staining. Protein bands were visualized using Clarity Western ECL Substrate (1705061, Bio-Rad) Western Blotting Detection Reagent.

### RNA sequencing analysis.

Total RNA was extracted from tumor tissues of mice with liver metastases. Total RNA samples were then submitted to Shanghai Bioprofile Co. Ltd. for preparation and construction of the mRNA library, followed by transcriptomic sequencing on the HiSeq X Ten System (Illumina). Cutadapt (v2.7) software (https://cutadapt.readthedocs.io/en/stable/) was used to filter the sequencing data to obtain a high-quality sequence (Clean Data) for further analysis. The clean reads were aligned to mouse GRCm39 genome assembly (v108.39) using HISAT2 (v2.2.1). Gene expression quantification was performed with HTSeq (v2.0.4; https://daehwankimlab.github.io/hisat2/manual/https://htseq.readthedocs.io/en/latest/). Differential expression analysis was performed using R package DESeq2 (version 1.38.3). Significantly differentially expressed genes were filtered out with *P* values less than 0.05 and fold change larger than 2. Heatmaps were generated by R package pheatmap (v1.0.12). Gene set enrichment analysis was performed by R package clusterProfiler (v4.6.2). RNA-Seq data generated in this study were deposited to the Gene Expression Omnibus (GEO) database under accession number GSE247303.

### Statistics.

Statistical analyses were carried out using GraphPad Prism 9 (GraphPad Software Inc.). One-way ANOVA with Kruskal-Wallis test was used to compare multiple groups. One-way ANOVA with Dunnett’s correct multiple-comparison test was used to compare multiple groups with the same control. Body weights of mice over time were compared using repeated-measurement ANOVA. The correlation between 2 variables was determined using standard Pearson’s correlation analysis. Wilcoxon’s matched-pairs signed rank test was used to test significance of difference between tumor uptake values of ^68^Ga-FAPI PET/CT imaging and ^18^F-FDG PET/CT imaging. The clinical outcomes of 2 groups were compared using χ^2^ test. A *P* value of less than 0.05 was considered statistically significant.

### Study approval.

Patient study was conducted in accordance with the principles of the Declaration of Helsinki and approved by the Ethics Committee of the FUSCC (approval ID 2004216025). Mouse studies were approved by the Research Ethical Committee of the FUSCC. All mouse studies were carried out in accordance with the requirements of the Animal Research Committee of Fudan University regarding the care and use of experimental animals in research (FUSCC-IACUC-S20210374).

### Data availability.

RNA-Seq data generated in this study were deposited to the GEO database under accession number GSE247303. All data supporting the findings of this study are available within the article and its supplemental material, including the Supporting Data Values file.

## Author contributions

ST and SS designed and guided the project. KL and WL designed, carried out, and analyzed most of the experiments. MQ, JZ, XX, HY, WT, and JW helped perform micro-MRI experiments and micro-PET imaging. JC contributed to the data analysis. Xinxiang Li, WG, and YS contributed to the clinical patient sample collection. KL and ST wrote the manuscript. Xiaoling Li, WL, and SS edited the manuscript. All authors read and agreed to the final version of the manuscript.

## Supplementary Material

Supplemental data

ICMJE disclosure forms

Unedited blot and gel images

Supporting data values

## Figures and Tables

**Table 2:**
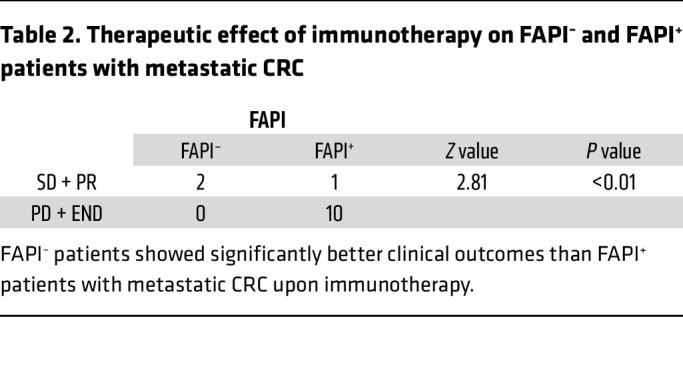
Therapeutic effect of immunotherapy on FAPI^–^ and FAPI^+^ patients with metastatic CRC

**Table 3:**
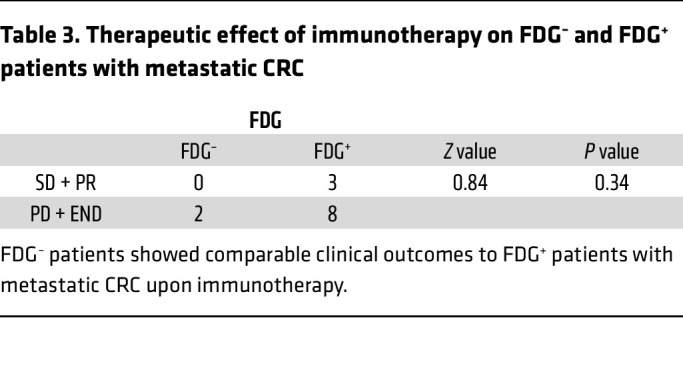
Therapeutic effect of immunotherapy on FDG^–^ and FDG^+^ patients with metastatic CRC

**Table 1:**
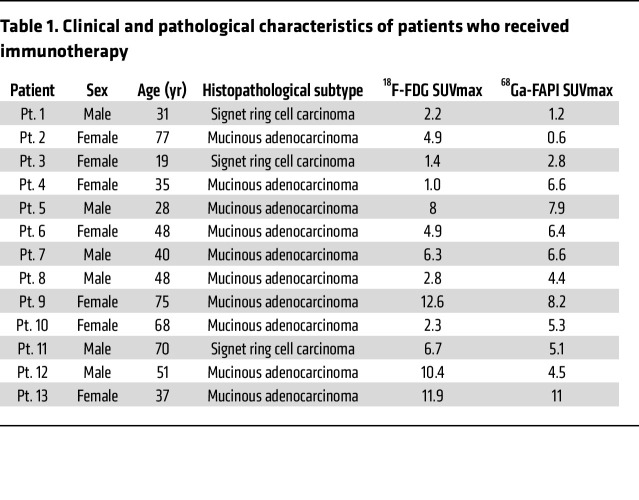
Clinical and pathological characteristics of patients who received immunotherapy

## Version 1 01/04/2024

In-Press Preview

## Version 2 02/15/2024

Electronic publication

## Version 3 02/21/2024

Added to authorship note ST and SS are co-corresponding authors.
