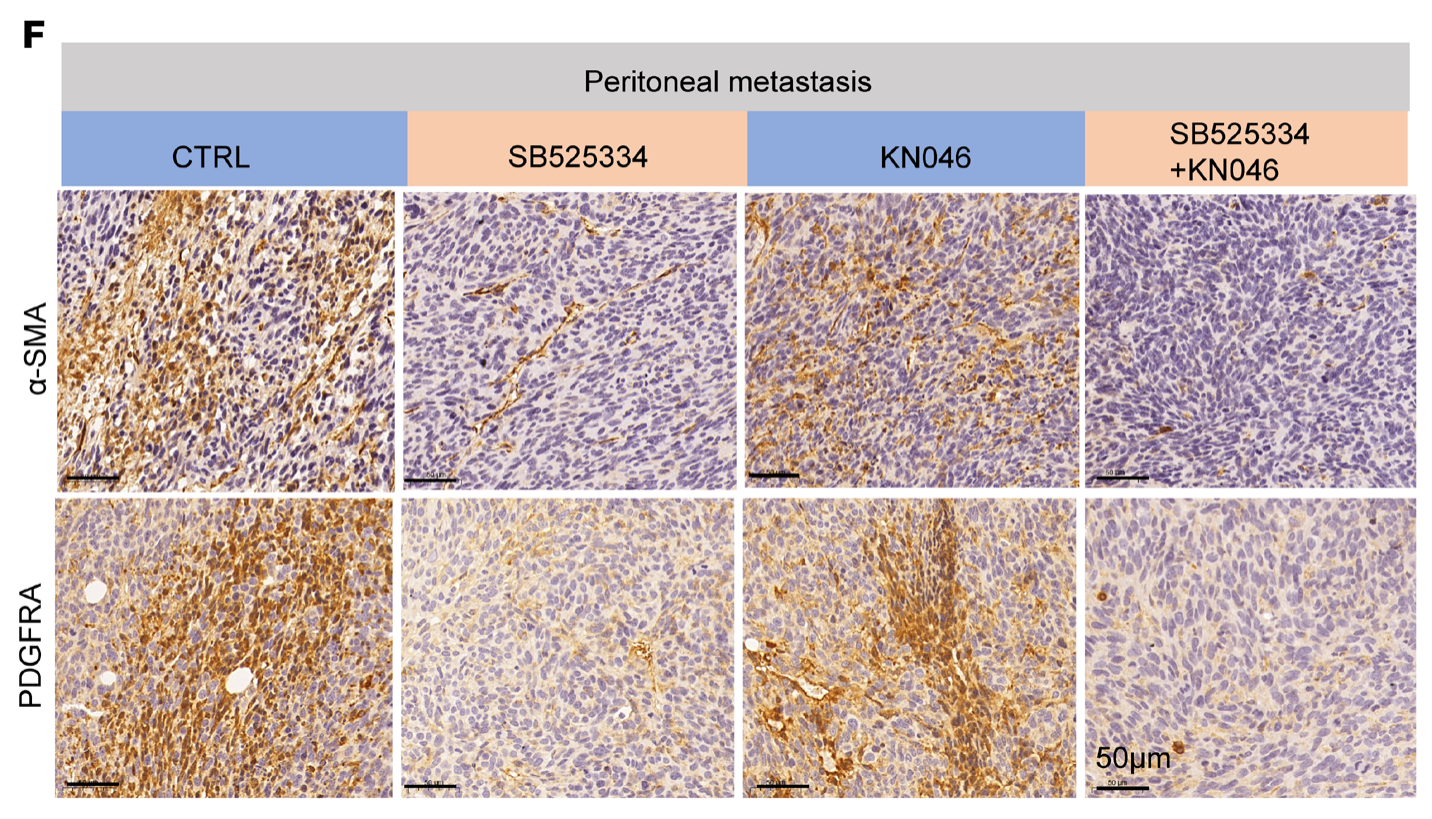# ^68^Ga-FAPI PET imaging monitors response to combined TGF-βR inhibition and immunotherapy in metastatic colorectal cancer

**DOI:** 10.1172/JCI181374

**Published:** 2024-04-15

**Authors:** Ke Li, Wei Liu, Hang Yu, Jiwei Chen, Wenxuan Tang, Jianpeng Wang, Ming Qi, Yuyun Sun, Xiaoping Xu, Ji Zhang, Xinxiang Li, Weijian Guo, Xiaoling Li, Shaoli Song, Shuang Tang

Original citation: *J Clin Invest*. 2024;134(4):e170490. https://doi.org/10.1172/JCI170490

Citation for this corrigendum: *J Clin Invest*. 2024;134(8):e181374. https://doi.org/10.1172/JCI181374

The authors recently became aware that in Figure 7F, an incorrect image was shown for α-SMA staining of the KN046-treated sample. The correct image, provided from the original source data, is shown below. The correction does not change the quantification that appears in Figure 1G. The HTML and PDF files have been updated with the correct information.

The authors regret the error.

## Figures and Tables

**Figure:**