# Supplementary material for: 68Ga-FAPI PET imaging monitors response to combined TGF-βR inhibition and immunotherapy in metastatic colorectal cancer
Source: J Clin Invest. 2024 Jan 4;134(4):e170490. doi: 10.1172/JCI170490 (PMC10866654; doi:10.1172/JCI170490)
Supplement: Supplemental data [file jci-134-170490-s012.pdf]

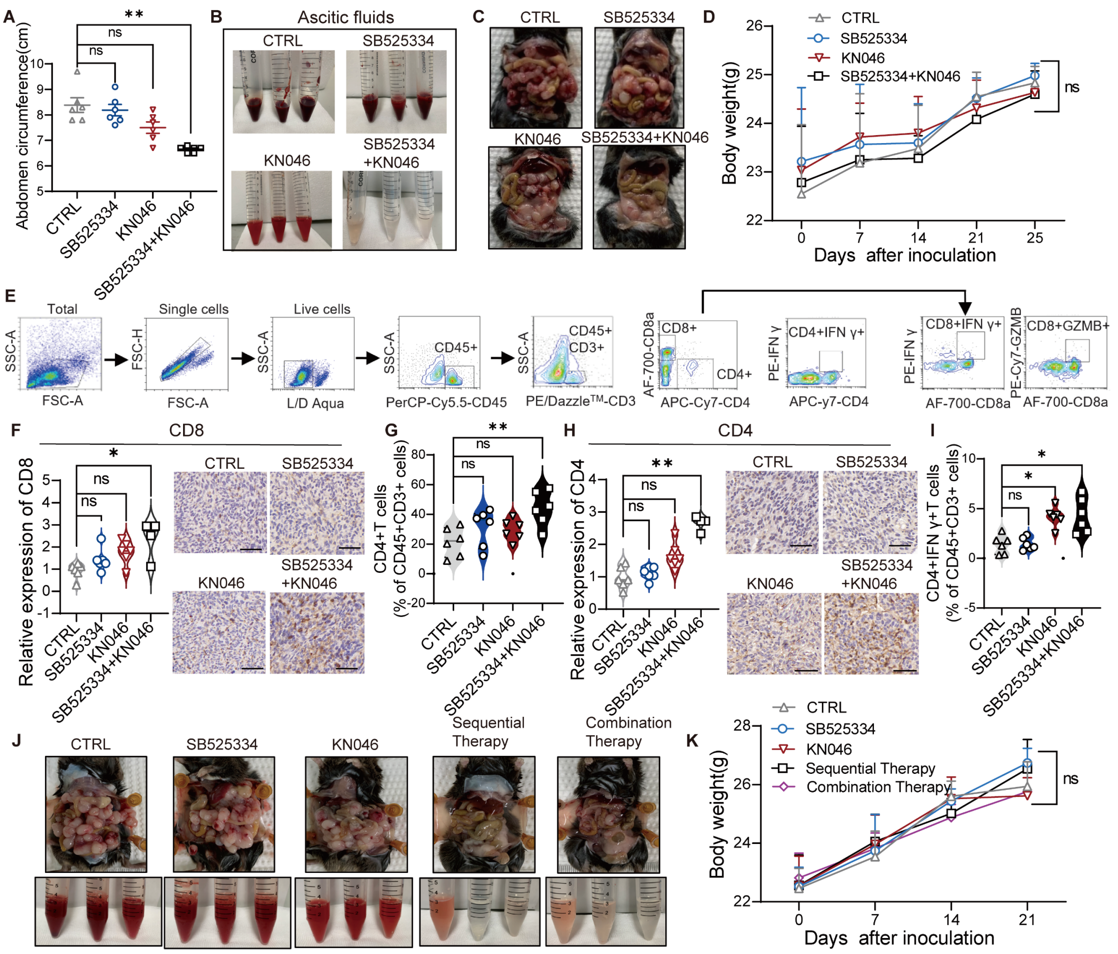

Supplementary Figure 1. TGF-β inhibition sensitizes mice with colorectal peritoneal metastases to KN046.

(A) Quantified abdomen circumference in mice with colorectal peritoneal metastasis after the four indicated treatments.

(B) Representative ascitic fluid of mice with colorectal peritoneal metastasis after the four indicated treatments (n=3 per group).

(C) Representative images of tumors in mice with colorectal peritoneal metastasis after the four indicated treatments.

(D) Body weight change of mice with colorectal peritoneal metastasis after the four indicated treatments.

(E) Representative flow cytometry gating strategy to quantify the numbers of various immune effector cell subsets in murine tumors.

(F) IHC staining and quantification of CD8 in peritoneal metastases of C57BL/6 mice of the four groups.

(G) Quantitative estimate of CD45+CD3+CD4+ T cell infiltration in peritoneal metastases after the four indicated treatments as determined using flow cytometry.

(H) IHC staining of CD4 and quantification in peritoneal metastases after the four indicated treatments.

(I) Quantitative estimate of CD4+IFNγ+ T cell infiltration in peritoneal metastases after the four indicated treatments as determined by flow cytometry.

(J) Representative tumors and ascitic fluid in mice with peritoneal metastasis after the indicated treatments.

(K) Body weight curve of the mice received the indicated treatments.

All numerical data are presented as mean ± SEM. *$p < 0.05$, **$p < 0.01$， ***$p < 0.001$ by one-way ANOVA with Dunnett correct multiple comparison test (A and F-I) and repeated measurement ANOVA test. ns, not significant.

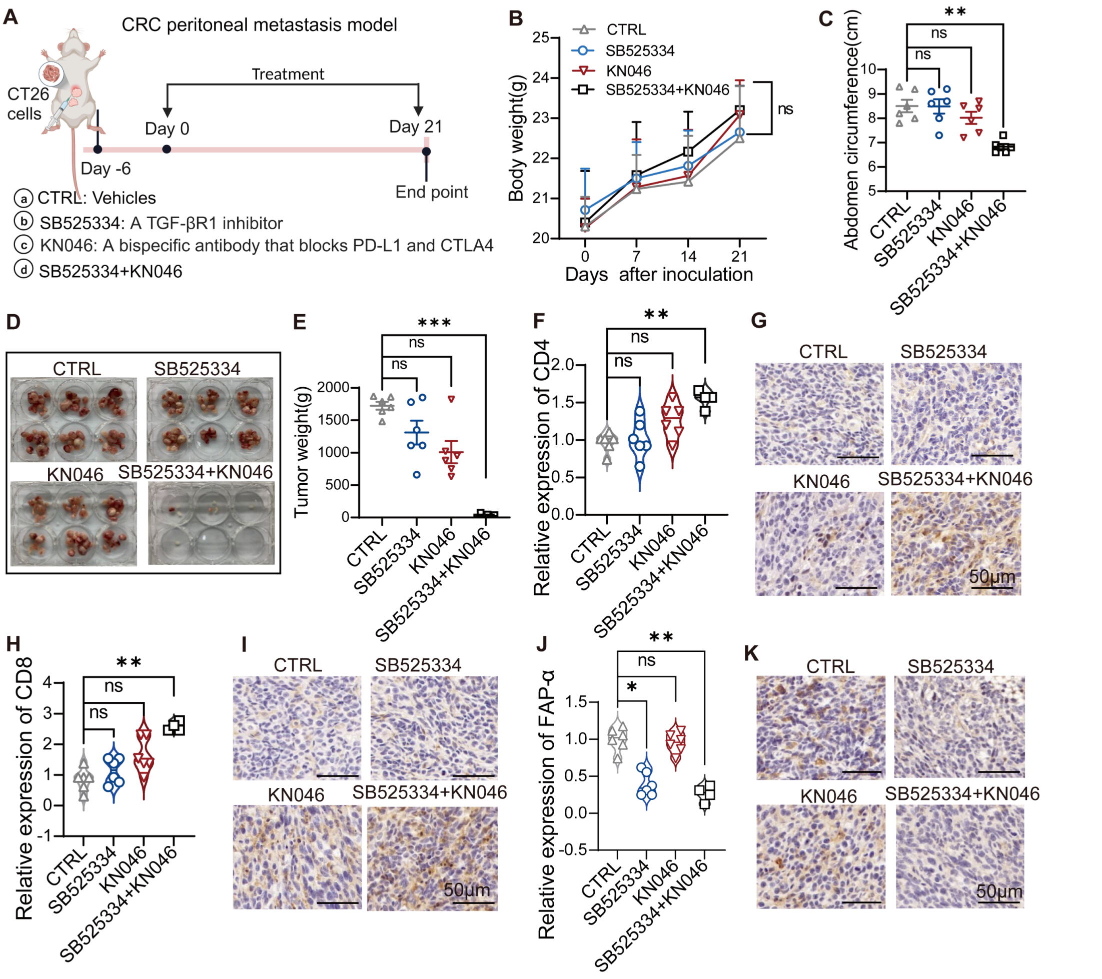

Supplementary Figure 2. ^{68}Ga-FAPI micro-PET/CT scans to assess tumor response to combined TGF-β receptor inhibitor and ICB KN046 in mice with CT26 colorectal peritoneal metastasis.

(A) Schematic representation of micro-PET/CT imaging and treatment strategies in BALB/c mice with CT26 peritoneal metastasis (four groups, n=6 per group). Created with BioRender.com.

(B) Body weight curves of mice under the indicated treatments.

(C) Abdomen circumference in mice with peritoneal metastasis under the indicated treatments.

(D) Representative tumors in mice with peritoneal metastasis after the indicated treatments.

(E) Tumor weight of mice with CT26 peritoneal metastasis after the indicated treatments.

(F–G) Quantified IHC staining of CD4 in tumors of mice with peritoneal metastasis after the indicated treatments.

(H–I) Quantified IHC staining of CD8 in tumors of mice with peritoneal metastasis after the indicated treatments.

(J–K) Quantified IHC staining of FAP-α in tumors of mice with peritoneal metastasis after the indicated treatments.

All numerical data are presented as mean ± SEM. *$p < 0.05$, **$p < 0.01$，***$p < 0.001$ by repeated measurement ANOVA test and one-way ANOVA with Dunnett correct multiple comparison test (C, E-F, H and J). ns, not significant.

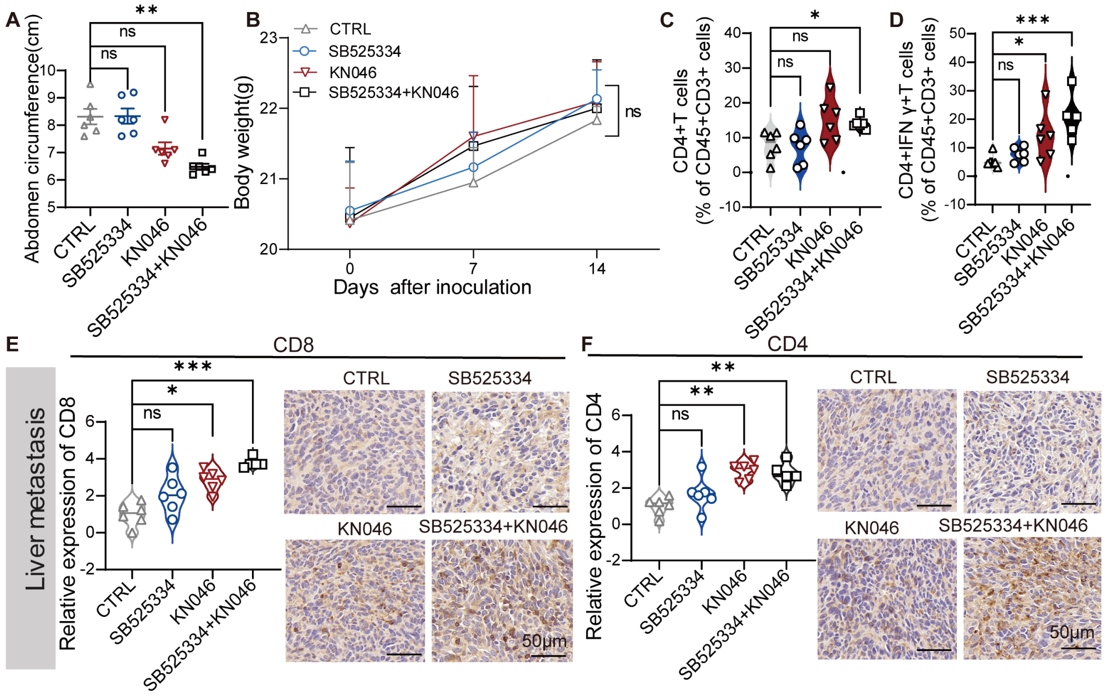

Supplementary Figure 3. TGF-β inhibition sensitizes mice with colorectal liver metastases to KN046.

(A) Quantified abdomen circumference in mice with MC38 liver metastasis after the four indicated treatments.

(B) Body weight changes of mice with colorectal liver metastasis after the four indicated treatments.

(C–D) Quantitative estimate of CD45+CD3+CD4+ T cell and CD4+IFN γ+ T cell infiltration in liver metastases after the four indicated treatments as determined using flow cytometry.

(E) IHC staining and quantification of CD8 in liver metastases after the four indicated treatments.

(F) IHC staining and quantification of CD4 in liver metastases after the four indicated treatments.

All numerical data are presented as mean ± SEM. $^*p < 0.05$, $^{**}p < 0.01$，$^{***}p < 0.001$ by one-way ANOVA with Dunnett correct multiple comparison test (A and C-F) and by repeated measurement ANOVA test (B). ns, not significant.

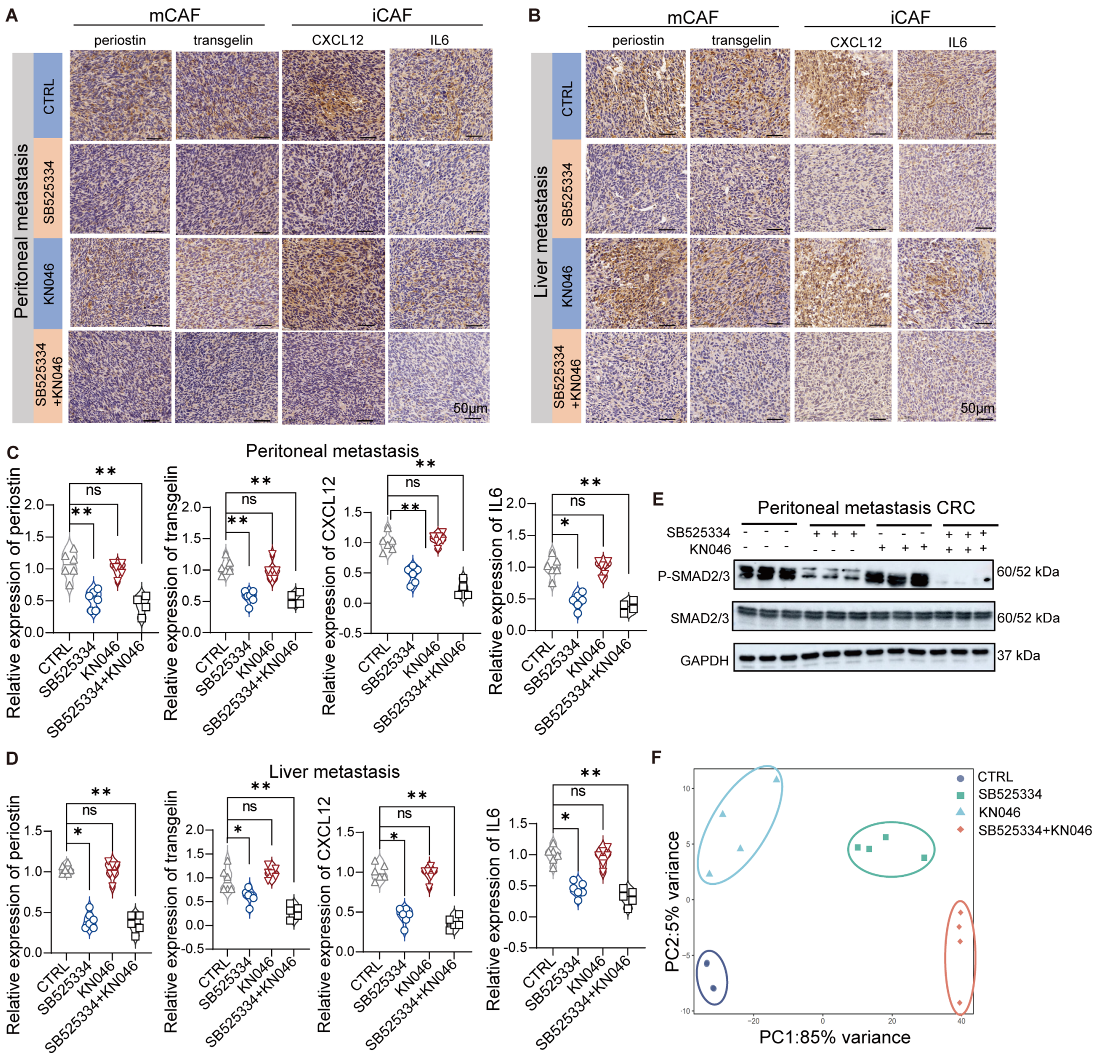

Supplementary Figure 4. TGF-β inhibition significantly reduced abundance of myCAF and iCAF in metastatic CRC.

(A–D) IHC staining and quantification of myCAF and iCAF makers in peritoneal and liver metastases of C57BL/6 mice after the indicated treatments.

(E) Immunoblotting analysis of SMAD2/3 phosphorylation, total SMAD2/3 and GAPDH levels in peritoneal metastases in mice after the indicated treatments.

(F) PCA of RNA-seq data in liver metastases of mice after the indicated treatments.

All numerical data are presented as mean ± SEM. *$p < 0.05$, **$p < 0.01$，***$p < 0.001$ by one-way ANOVA with Dunnett correct multiple comparison test (C and D). ns, not significant.
