# Supplementary figures and images for: 68Ga-FAPI PET imaging monitors response to combined TGF-βR inhibition and immunotherapy in metastatic colorectal cancer
Source: J Clin Invest. 2024 Jan 4;134(4):e170490. doi: 10.1172/JCI170490 (PMC10866654; doi:10.1172/JCI170490)

Full unedited gel for Figure S4E

Peritoneum metastasis CRC

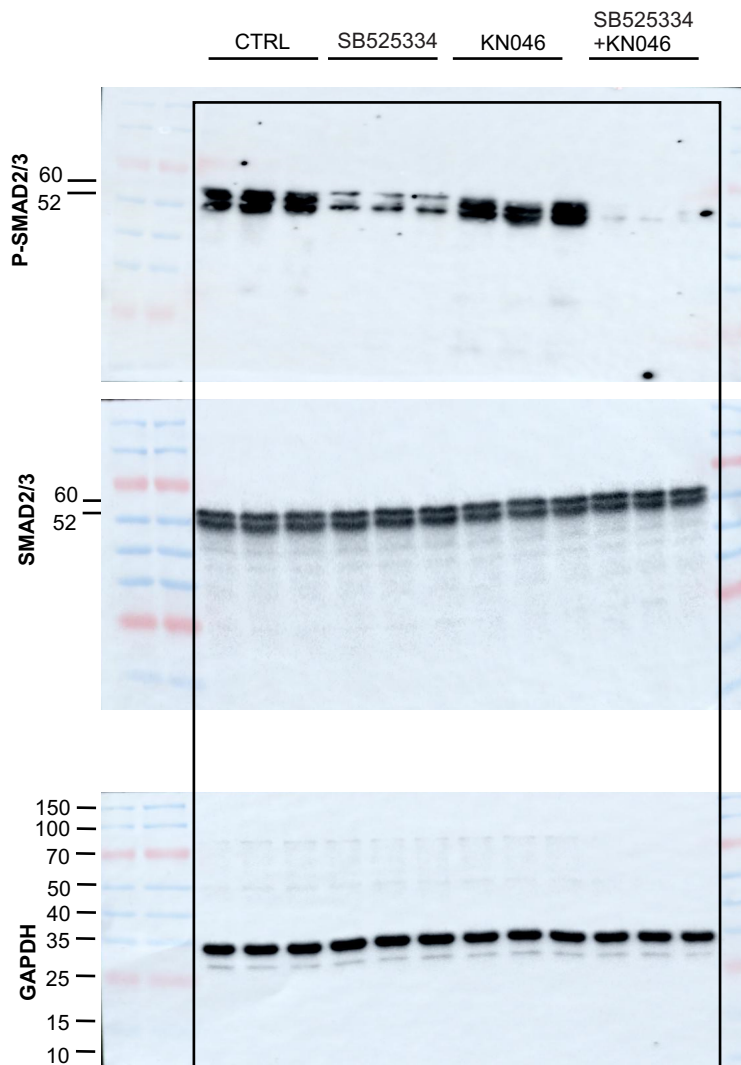

Supplement: Unedited blot and gel images [file jci-134-170490-s014.pdf]
